# ECG-XPLAIM: eXPlainable Locally-adaptive Artificial Intelligence Model for arrhythmia detection from large-scale electrocardiogram data

**DOI:** 10.3389/fcvm.2025.1659971

**Published:** 2025-10-16

**Authors:** Panteleimon Pantelidis, Samuel Ruipérez-Campillo, Julia E. Vogt, Alexios Antonopoulos, Ioannis Gialamas, George E. Zakynthinos, Michael Spartalis, Polychronis Dilaveris, Jose Millet, Theodore G. Papaioannou, Evangelos Oikonomou, Gerasimos Siasos

**Affiliations:** ^1^ 3rd Department of Cardiology, National and Kapodistrian University of Athens, Athens, Greece; ^2^ Department of Computer Science, ETH Zurich, Zurich, Switzerland; ^3^ 1st Department of Cardiology, National and Kapodistrian University of Athens, Athens, Greece; ^4^ ITACA Institute, Universitat Politecnica de Valencia, Valencia, Spain

**Keywords:** arrhythmia, electrocardiogram, artificial intelligence, deep learning, machine learning, cardiac signals, explainability

## Abstract

**Background:**

Timely and accurate detection of arrhythmias from electrocardiograms (ECGs) is crucial for improving patient outcomes. While artificial intelligence (AI)-based ECG classification has shown promising results, limited transparency and interpretability often impede clinical adoption.

**Methods:**

We present ECG-XPLAIM, a novel deep learning model dedicated to ECG classification that employs a one-dimensional inception-style convolutional architecture to capture local waveform features (e.g., waves and intervals) and global rhythm patterns. To enhance interpretability, we integrate Grad-CAM visualization, highlighting key waveform segments that drive the model's predictions. ECG-XPLAIM was trained on the MIMIC-IV dataset and externally validated on PTB-XL for multiple arrhythmias, including atrial fibrillation (AFib), sinus tachycardia (STach), conduction disturbances (RBBB, LBBB, LAFB), long QT (LQT), Wolff-Parkinson-White (WPW) pattern, and paced rhythm detection. We evaluated performance using sensitivity, specificity, and area under the receiver operating characteristic curve (AUROC), and benchmarked against a simplified convolutional neural network, a two-layer gated recurrent unit (GRU), and an external, pre-trained, ResNet-based model.

**Results:**

Internally (MIMIC-IV), ECG-XPLAIM achieved high diagnostic performance (sensitivity, specificity, AUROC > 0.9) across most tasks. External evaluation (PTB-XL) confirmed generalizability, with metric values exceeding 0.95 for AFib and STach. For conduction disturbances, macro-averaged sensitivity reached 0.90, specificity 0.95, and AUROC 0.98. Performance for LQT, WPW, and pacing rhythm detection was 0.691/0.864/0.878, 0.773/0.973/0.895, and 0.96/0.988/0.993 (sensitivity/specificity/AUROC), respectively. Compared to baseline models, ECG-XPLAIM offered superior performance across most tests, and improved sensitivity over the external ResNet-based model, albeit at the cost of specificity. Grad-CAM revealed physiologically relevant ECG segments influencing predictions and highlighted patterns of potential misclassification.

**Conclusion:**

ECG-XPLAIM combines high diagnostic performance with interpretability, addressing a key limitation in AI-driven ECG analysis. The open-source release of ECG-XPLAIM's architecture and pre-trained weights encourages broader adoption, external validation, and further refinement for diverse clinical applications.

## 1 Introduction

Accurate arrhythmia detection from electrocardiogram (ECG) recordings is crucial for early intervention, particularly for life-threatening conditions such as atrial fibrillation, conduction disturbances, and other arrhythmic syndromes. If left undiagnosed or untreated, these conditions can lead to severe complications that adversely affect patient morbidity and mortality. For instance, atrial fibrillation increases the risk of stroke fivefold (1), while Wolff-Parkinson-White syndrome, long QT syndrome, and other arrhythmic disorders markedly raise susceptibility to fatal cardiac events (2). Despite the importance of prompt diagnosis, ECG interpretation is highly specialized; underdiagnosis rates exceeding 50% are reported among non-cardiologists, varying with disease, population, and clinical setting (3). This highlights the pressing need for automated diagnostic tools that can support clinicians, reduce human error, and improve arrhythmia detection rates.

Artificial intelligence (AI) and deep learning (DL) have emerged as powerful tools capable of automating ECG analysis, achieving high accuracy even in detecting atypical cases. These models can recognize complex patterns in ECG signals, often outperforming rule-based algorithms and, in some scenarios, surpassing human expertise (4–7). Nonetheless, significant challenges remain. Existing DL models frequently overlook the unique attributes of ECG signals—which exhibit both repetitive waveforms and global rhythmic patterns—requiring both local (wave- and interval-level) and global (rhythm-level) analysis (8, 9). Additionally, many proposed methods are closed-source, limiting adaptation to specialized clinical applications and generalization across diverse populations (10, 11). Another critical concern is the interpretability of AI-driven decisions; clinicians must understand model reasoning before relying on these tools for patient care (12–14). Explainable AI (XAI) techniques, including Grad-CAM (15, 16), highlight the salient ECG waveform features that contribute most to the model's decisions, thus improving interpretability. However, relatively few ECG-focused architectures combine robust performance with built-in explainability.

In this study, we introduce ECG-XPLAIM—an eXPlainable, Locally-adaptive Artificial Intelligence Model designed for ECG classification. By leveraging a deep inception-style convolutional architecture (17), ECG-XPLAIM is well-suited for time-series data analysis where capturing temporal dependencies is essential. The model further integrates XAI principles (13), aligning its decision-making process with the clinical reasoning that underpins ECG interpretation. Trained on large-scale ECG datasets to ensure scalability and broad clinical applicability, ECG-XPLAIM is also released as open-source, complete with pre-trained weights. The open framework facilitates external validation, transfer learning, and customization for specialized tasks. Overall, our work aims to advance interpretable machine learning in healthcare, offering a framework that balances high diagnostic performance with transparency and adaptability, thereby enhancing trust in AI-driven ECG diagnostics.

## 2 Materials and methods

### 2.1 Study population and data sources

**Figure 1:**
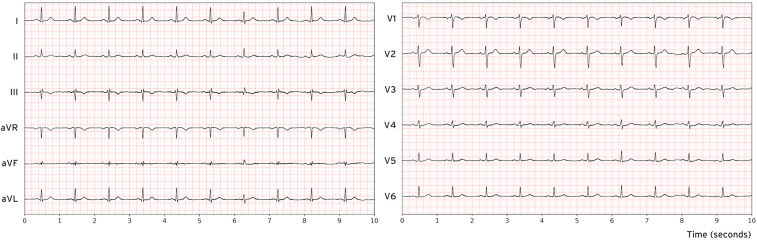
Visualization of a 12-lead normal electrocardiogram sample from the MIMIC-IV database.

We employed two large-scale, publicly available ECG datasets for model development and evaluation: MIMIC-IV and PTB-XL (18–20). MIMIC-IV contains over 800,000 12-lead ECG recordings, while PTB-XL comprises more than 25,000 12-lead ECG records, each sampled at 500 Hz for a uniform duration of 10 s (Figure 1). The raw waveform data were used without additional preprocessing, aside from replacing missing values with zeros. To evaluate the impact of conventional ECG preprocessing, additional experiments applied a 0.5–40 Hz Butterworth bandpass filter and a 50 Hz notch filter prior to training and inference, while keeping all other parameters identical (see Supplementary Section S4). Demographic and patient-specific metadata were deliberately excluded from the training process to focus exclusively on ECG waveforms and to minimize bias related to patient characteristics.

### 2.2 Diagnostic labels and outcome selection

We defined five classification tasks, each of them corresponding to a clinically significant arrhythmic category (Table 1). The first task (tachycardia—TACHY) involved distinguishing atrial fibrillation (AFib) from sinus tachycardia (STach). The second task focused on detecting conduction disturbances (CD), encompassing right bundle branch block (RBBB), left bundle branch block (LBBB), and left anterior fascicular block (LAFB). The remaining tasks targeted the identification of long QT (LQT), the detection of Wolff-Parkinson-White (WPW) pattern, and the recognition of paced (PACE) rhythms.

**Table 1:** Arrhythmia classification tasks and definitions.

Task	Diagnostic labels	Details
TACHY (Tachyarrhythmias)	AFib vs. STach vs. Neg^a^	Differentiates AFib, characterized by irregular RR intervals and absent P-waves, from STach, which exhibits regular RR intervals with elevated heart rates. The Neg class includes ECGs that do not show these tachyarrhythmias.
CD (Conduction Disturbances)	RBBB vs. LBBB vs. LAFB vs. Neg^a^	Detects bundle branch blocks based on QRS complex morphology and duration. RBBB is marked by a prolonged QRS (>120 ms) with an rSR’ pattern in V1, LBBB by a broad QRS with deep S waves in V1, and LAFB by left-axis deviation and qR pattern in aVL. The Neg group excludes these conditions.^b^
LQT (Long QT)	LQT vs. Neg^a^	Identifies prolonged QT intervals, measured using standard correction formulas.
WPW (Wolff-Parkinson-White) pattern	WPW pattern vs. Neg^a^	Detects pre-excitation patterns characterized by short PR intervals, delta waves, and wide QRS complexes, which indicate an accessory conduction pathway.
PACE (Paced rhythm)	Paced rhythm vs. Neg^a^	Identifies cardiac pacing (atrial and/or ventricular), marked by pacemaker spikes preceding P-waves and/or QRS complexes, respectively.

AFib, atrial fibrillation; LAFB, left anterior fascicular block; LBBB, left bundle branch block; LQT, long QT; RBBB, right bundle branch block; STach, sinus tachycardia; WPW, Wolff-Parkinson-White pattern.

^a^ Neg, the negative class within each task does not necessarily represent normal ECGs—it may include other non-target arrhythmias or abnormalities.

^b^ Left posterior fascicular block (LPFB) was excluded due to limited representation in the dataset.

Labels were derived from structured diagnostic reports and automated ECG annotations provided within the source databases (a detailed list of key terms is presented in the Supplementary Section S1). Negative samples for each task were defined by the absence of the corresponding arrhythmic condition but could include other pathologies unrelated to the primary label. Overlapping conditions (for example, AFib coexisting with LQT or WPW overlapping with LBBB) were not excluded to reflect the complexity of real-world ECG interpretations. A manual review of the entire PTB-XL test subset, and an additional random 10% of the MIMIC-IV dataset was performed by expert clinicians to validate labeling accuracy. Agreement with database-provided labels was quantified using Cohen's kappa with bootstrapped CIs (Supplementary Section S5).

### 2.3 Model design and explainability mechanism

**Figure 2:**
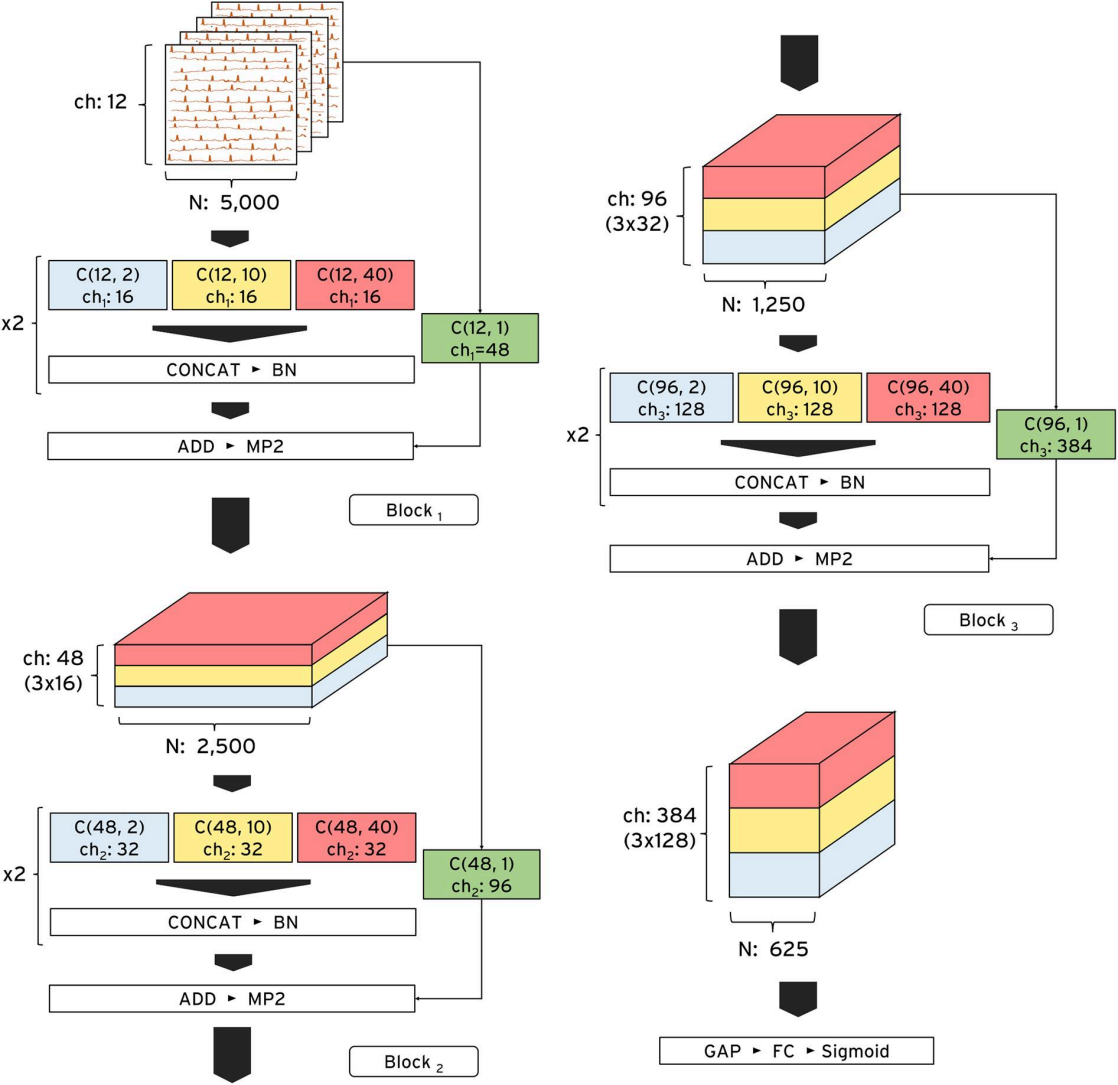
ECG-XPLAIM architecture. The model consists of three sequential Inception blocks optimized for time-series ECG analysis. Each block contains two repeated Inception modules, each incorporating three parallel one-dimensional convolutional filters with multiple channels. These filters, with receptive field lengths of 2, 10, and 40 data points, capture fine-grained, intermediate, and long-range ECG features, respectively. At the end of each module, the outputs are concatenated and passed to the next layer. The input dimensions are 12 (number of leads) * 5,000 (10-s recordings at a 500 Hz sampling rate), while the output depends on the classification task. ADD, addition layer; BN, batch normalization layer; C, one-dimensional convolutional layer; CONCAT, concatenation layer; ch, channels; FC, fully connected (dense) layer; GAP, global average pooling layer; MP2, max pooling layer with pool size 2; N, number of data points; Sigmoid, Sigmoid activation function.

We developed ECG-XPLAIM using a custom Inception-style convolutional neural network (CNN) architecture, designed for time-series analysis (17, 21). This framework captures both local waveform features (e.g., P, QRS, and T waves and intervals) and global rhythm patterns, such as irregularities that underlie arrhythmias. The model comprises three residual blocks, each containing two one-dimensional Inception modules. Within each module, three convolutional kernels of lengths 2, 10, and 40 data points (corresponding to 4, 20, and 80 ms at 500 Hz sampling frequency) operate in parallel. The outputs of these parallel paths are concatenated before being fed into the subsequent layer. By incorporating skip connections, we preserved signal integrity and avoided feature degradation as the network depth increased (Figure 2). The receptive field expands progressively, roughly doubling after each block, which allows ECG-XPLAIM to detect both brief waveform and broader rhythm disturbances. This emphasizes high sensitivity for transient lesions while maintaining robust generalization across diverse ECG patterns.

To enhance interpretability, we integrated a customized one-dimensional Gradient-weighted Class Activation Mapping (Grad-CAM) mechanism tailored for multi-lead ECGs (15). This approach generates activation heatmaps that highlight the waveform regions most influential for the model's predictions. By aggregating lead-specific Grad-CAM maps across all 12 leads, ECG-XPLAIM provides a comprehensive view of its decision-making. We overlayed these heatmaps on the raw ECG traces to underline clinically relevant features such as the presence or absence of P-wave and their morphology, QRS complex widening, and RR interval variations.

### 2.4 Training and evaluation strategies

We trained ECG-XPLAIM on the MIMIC-IV dataset and then evaluated internally on independent cohorts from the same dataset, while also testing externally on PTB-XL. To address class imbalance, the maximum number of samples per class was capped at 50,000, except for WPW detection, where data scarcity necessitated a 1:2 positive-to-negative ratio (600 vs. 1,220 samples). For AFib vs. STach vs. Negative, 50,000 samples were used per class. For RBBB, LBBB, LAFB, and Negative, 25,000 were assigned to each category. For LQT vs. Negative, 39,000 samples per class were used, and for PACE vs. Negative, 30,000 per class were retained. Distinct training, validation, and test subsets were prepared to avoid overlapping. As an illustrative experiment, we further fine-tuned the pre-trained MIMIC-IV model on varying fractions of PTB-XL (0%, 5%, 10%, 20%, 50%) and evaluated it on the remaining data (Supplementary Section S6). This setup is included to demonstrate feature transferability, while it is not part of our primary claims, since it does not fulfill the requirement for independence of the external dataset (22).Training was performed in mini-batches using TensorFlow/Keras on an NVIDIA L4 GPU (24GB VRAM) for 100 epochs without early stopping. We adopted the Adaptive Moment Estimation (ADAM) optimizer (23), with an initial learning rate of 0.01, subject to exponential decay (0.95 per epoch). The batch size was 128 for most tasks but reduced to 32 for WPW detection due to fewer positive samples. We selected the optimal checkpoint based on validation metrics during training to minimize overfitting.

### 2.5 Diagnostic performance and statistical analysis

We evaluated model performance using sensitivity (recall), specificity, and area under the receiver operating characteristic curve (AUROC), estimating 95% confidence intervals (CIs) for each metric. Internal assessment took place on the MIMIC-IV test set, whereas external evaluation was performed on the PTB-XL cohort, ensuring no overlap with training data. CIs for sensitivity and specificity were computed using the Clopper-Pearson exact method (24), and AUROC CIs were derived via DeLong's method (25). We also evaluated task-wise operating points suitable for screening (maximizing sensitivity) and diagnostic confirmation (maximizing specificity), by scanning model decision thresholds from 0.0 to 1.0, at 0.1 increments, and reporting macro-averaged sensitivity/specificity pairs.

To establish benchmark comparisons, we trained two deep learning baselines under identical conditions. First, we implemented a conventional one-dimensional CNN model (vanilla CNN) with three standard convolutional layers followed by batch normalization and max pooling. Second, we applied a more advanced, double-layered, gated recurrent unit (GRU) architecture, designed for time-series feature extraction (implementation details are provided in Supplementary Section S2) (26). We also compared against an external, pre-trained, ResNet-based deep network that had been previously validated for 12-lead ECG classification (27). This external model was adapted to match our sampling frequency, lead configuration, and data settings. As the commonly supported diagnostic categories were only four (AFib, STach, RBBB, and LBBB), comparisons were restricted to these conditions.

We performed pairwise statistical analyses between ECG-XPLAIM and the counterpart models, across all classification tasks, to identify significant differences in performance. We used McNemar's test to compare model sensitivity and specificity (28, 29), and applied bootstrap resampling (*n* = 1,000) to compare AUROC differences (30), employing a significance threshold of 0.05. To assess interpretability, we generated Grad-CAM heatmaps for correctly classified and misclassified recordings, examining which waveform components informed the model's predictions. We compared these heatmaps across all tasks, offering insight into potential biases and failure modes.

## 3 Results

### 3.1 Diagnostic performance evaluation

#### 3.1.1 Internal evaluation

ECG-XPLAIM demonstrated robust classification performance across all arrhythmia detection tasks in the MIMIC-IV test set (Table 2). For tachyarrhythmia classification, it achieved a sensitivity of 0.897 (95% CI: 0.876–0.915) for AFib, and 0.95 (95% CI: 0.935–0.963) for STach. Specificity and AUROC values were greater or equal to 0.94 and 0.98, respectively. When detecting conduction disturbances (RBBB, LBBB, and LAFB), the model yielded macro-averaged sensitivity, specificity, and AUROC of 0.941, 0.972, and 0.993, respectively, demonstrating consistent performance across these abnormalities.

**Table 2:** Diagnostic performance of ECG-XPLAIM in internal and external evaluation.

Task	Internal evaluation (MIMIC-IV)			External evaluation (PTB-XL)		
	Sensitivity	Specificity	AUROC	Sensitivity	Specificity	AUROC
TACHY						
AFib	0.897 (0.876, 0.915)	0.939 (0.928, 0.949)	0.976 (0.97, 0.981)	0.954 (0.942, 0.964)	0.964 (0.955, 0.971)	0.988 (0.984, 0.991)
STach	0.95 (0.935, 0.963)	0.978 (0.97, 0.984)	0.995 (0.992, 0.997)	0.956 (0.94, 0.969)	0.974 (0.967, 0.979)	0.991 (0.988, 0.994)
macro-avg	0.924	0.958	0.987	0.955	0.969	0.99
CD						
RBBB	0.95 (0.938, 0.961)	0.974 (0.967, 0.98)	0.994 (0.992, 0.997)	0.996 (0.986, 1)	0.966 (0.959, 0.972)	0.994 (0.992, 0.997)
LBBB	0.945 (0.929, 0.958)	0.982 (0.976, 0.986)	0.995 (0.993, 0.998)	0.99 (0.977, 0.997)	0.927 (0.918, 0.936)	0.99 (0.987, 0.993)
LAFB	0.928 (0.913, 0.941)	0.961 (0.953, 0.968)	0.989 (0.985, 0.992)	0.714 (0.691, 0.736)*	0.962 (0.954, 0.97)	0.946 (0.939, 0.953)
macro-avg	0.941	0.972	0.993	0.9	0.952	0.977
LQT	0.93 (0.912, 0.945)	0.897 (0.876, 0.915)	0.969 (0.962, 0.977)	0.691 (0.596, 0.776)*	0.864 (0.785, 0.922)	0.878 (0.835, 0.922)
WPW	0.99 (0.946, 1)	0.95 (0.887, 0.984)	0.992 (0.98, 1)	0.773 (0.662, 0.862)*	0.973 (0.907, 0.997)	0.895 (0.846, 0.944)
PACE	0.927 (0.909, 0.942)	0.983 (0.973, 0.99)	0.985 (0.979, 0.99)	0.96 (0.928, 0.981)	0.988 (0.965, 0.998)	0.993 (0.985, 1)

AFib, atrial fibrillation; AUROC, area under the receiver operating characteristic; CD, conduction disturbance task; LAFB, left anterior fascicular block; LBBB, left bundle branch block; LQT, long QT; Macro-avg, macro-averaged metric; PACE, Paced rhythm; RBBB, right bundle branch block; STach, Sinus tachycardia; TACHY, Tachycardia task; WPW, Wolff-Parkinson-White pattern.

Metrics are reported with 95% confidence intervals; The exact number of samples per class for each task is provided in the Supplementary Material; Metrics marked with an asterisk (*) indicate values below 0.9 with 95% confidence.

**Figure 3:**
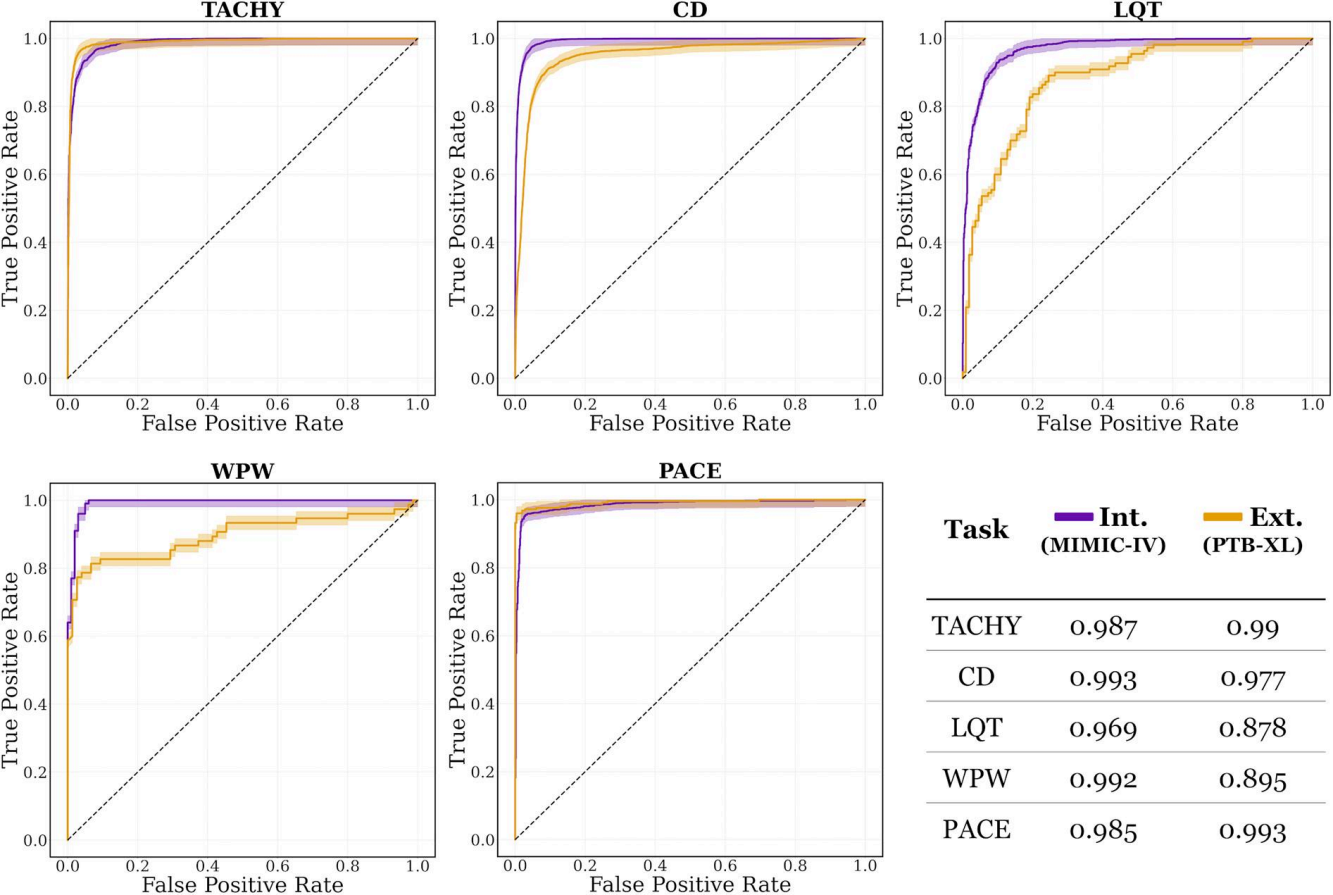
Area under the receiver operating characteristic (AUROC) curves for internal (Int.) and external (Ext.) evaluations, on MIMIC-IV and PTB-XL datasets, respectively. The curves are plotted with their corresponding 95% confidence intervals. CD, conduction disturbance task (includes right and left bundle branch block, as well as left anterior fascicular block); LQT, long QT detection task; PACE, paced rhythm task; TACHY, tachycardia task (includes atrial fibrillation and sinus tachycardia); WPW, Wolff-Parkinson-White pattern.

ECG-XPLAIM achieved a sensitivity of 0.93 (95% CI: 0.912–0.945) and specificity of 0.897 (95% CI: 0.876–0.915) for LQT detection, with an AUROC of 0.969 (95% CI: 0.962–0.977). For WPW pattern, it attained a sensitivity of 0.99 (95% CI: 0.946–1) and specificity of 0.95 (95% CI: 0.887–0.984), translating to an AUROC of 0.992 (95% CI: 0.980–1). PACE detection yielded a sensitivity of 0.927 (95% CI: 0.909–0.942) and specificity of 0.983 (95% CI: 0.973–0.99), with an AUROC of 0.985 (95% CI: 0.979–0.99). Overall, macro-averaged AUROC values exceeded 0.96 for all tasks in the internal evaluation, as illustrated in Figure 3.

#### 3.1.2 External evaluation

Evaluation on PTB-XL, which was not used during model development, confirmed the strong generalizability of ECG-XPLAIM for most arrhythmias (Table 2). In AFib and STach classification, sensitivity values were 0.954 (95% CI: 0.942–0.964) and 0.956 (95% CI: 0.940–0.969), respectively, with specificity of 0.964 (95% CI: 0.955–0.971) and 0.974 (95% CI: 0.967–0.979), and AUROC of 0.988 (95% CI: 0.984–0.991) and 0.991 (95% CI: 0.988–0.994). Conduction disturbance detection remained similarly robust, with RBBB identified at a sensitivity of 0.996 (95% CI: 0.986–1) and specificity of 0.966 (95% CI: 0.959–0.972). Although LBBB achieved a sensitivity of 0.99 (95% CI: 0.977–0.997), its specificity was lower at 0.927 (95% CI: 0.918–0.936). LAFB exhibited a reduced sensitivity of 0.714 (95% CI: 0.691–0.736), but maintained a high specificity of 0.962 (95% CI: 0.954–0.970).

LQT detection showed a modest decline in performance compared to internal evaluation, with a sensitivity of 0.691 (95% CI: 0.596–0.776), specificity of 0.864 (95% CI: 0.785–0.922), and an AUROC of 0.878 (95% CI: 0.835–0.922). Detection of WPW pattern also revealed a drop in sensitivity to 0.773 (95% CI: 0.662–0.862), with specificity remaining high at [0.973 (95% CI: 0.907–0.997)]. Paced rhythm detection achieved a sensitivity of 0.96 [95% CI: 0.928–0.981)], specificity of 0.988 (95% CI: 0.965–0.998), and AUROC of 0.993 (95% CI: 0.985–1).

Overall, while certain conditions (notably LQT and WPW) showed reduced sensitivity on the external dataset, AUROC values persisted between 0.88 and 0.99 across tasks, confirming the model's strong generalization capabilities (Figure 3).

#### 3.1.3 Supplementary analyses

We explored the effect of preprocessing (bandpass, notch), showing minimal or adverse impact on performance (Supplementary Table S3). For example, pacing detection sensitivity fell when filtering likely attenuated pacing spikes. Inter-observer agreement confirmed excellent label quality (Cohen's *α* ≥ 0.99 across all classes; Supplementary Table S4). Transfer learning experiments demonstrated that fine-tuning with small PTB-XL fractions could improve sensitivity for some tasks (e.g., WPW), but at the cost of specificity for some classes such as LQT, consistent with overfitting (Supplementary Table S5, Supplementary Figure S3). Threshold scans revealed balanced operating points at thresholds 0.4–0.6 for specific tasks, such as TACHY, CD and PACE, while for the rest, particularly for LQT, context-dependent trade-offs were illustrated (Supplementary Table S6).

#### 3.1.4 Explainability analysis

**Figure 4:**
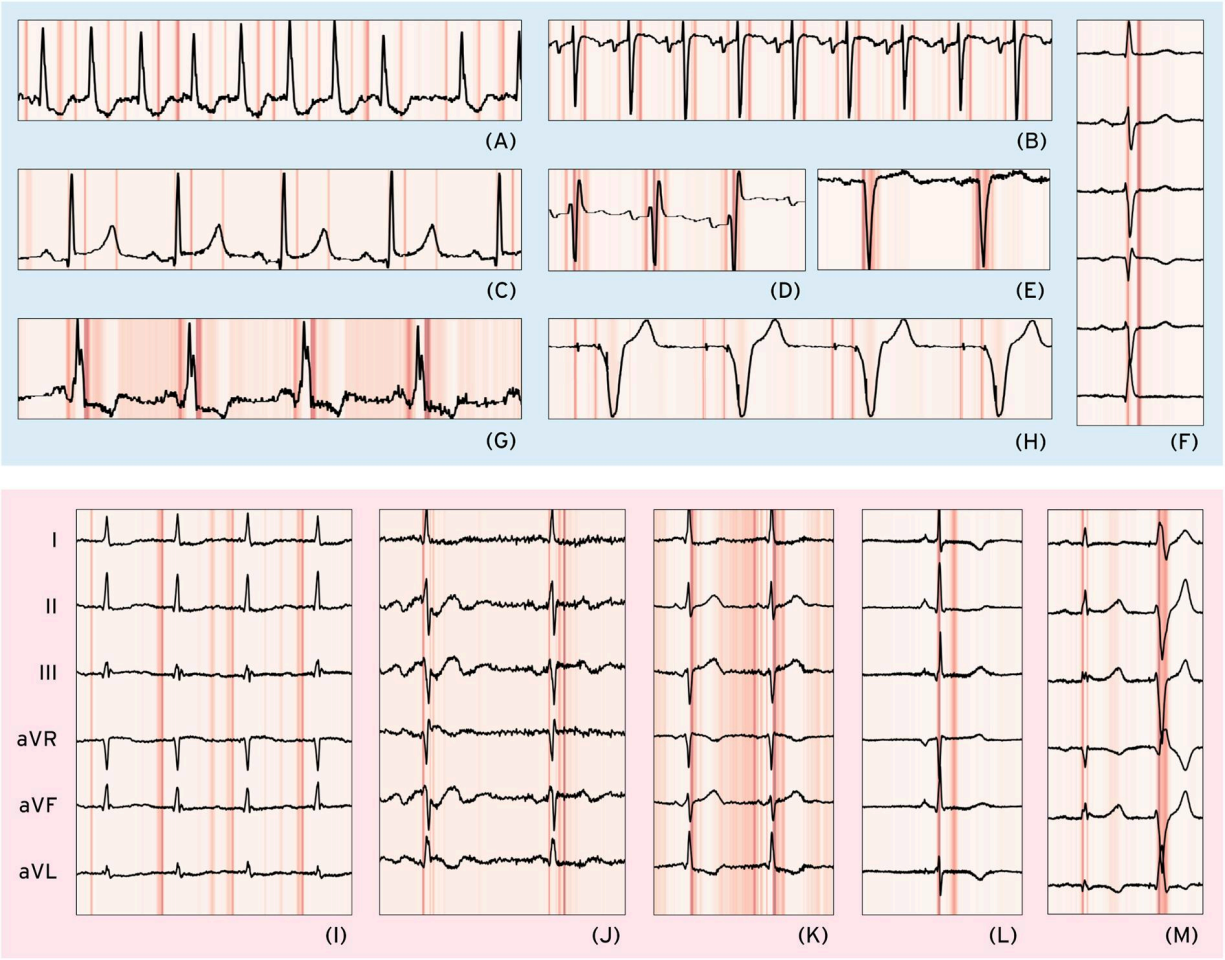
Selected correct and misclassified cases. Correctly classified cases (light blue background, upper half): **(A)** atrial fibrillation (AFib)—Lead II, **(B)** sinus tachycardia—Lead V1, **(C)** long QT (LQT)—Lead II, **(D)** right bundle branch block—Lead V1, **(E)** left bundle branch block—Lead V1, **(F)** left anterior fascicular block (LAFB)—Limb leads, **(G)** Wolff–Parkinson–White (WPW) pattern—Lead II, **(H)** paced rhythm (PACE)—Lead V1. Misclassified cases (light red background, lower half) with only limb leads shown: **(I)** AFib—false Positive due to coexisting first-degree AV block (I-AVB), possibly leading to P-wave misinterpretation, **(J)** LQT—false Negative possibly due to unclear/biphasic T-waves preventing accurate QT interval measurement, **(K)** WPW—False Negative where multiple points of interest before the QRS complex cause potential misinterpretation, **(L)** PACE—False Positive due to possible misclassification of a narrow QRS as a pacing spike, **(M)** LAFB—False Positive where a premature ventricular complex is possibly mistaken for a normal beat, leading to axis misinterpretation as LAFB-like.

We performed Grad-CAM-based analysis to visualize the waveform regions that informed ECG-XPLAIM's classification decisions. The model highlighted clinically meaningful features for each arrhythmia, while misclassifications offered insights into the model's potential attention biases, comprising focus on specific wave morphology abnormalities and interval duration deviations. Figure 4 presents single- or few-lead visualizations of selected correctly and incorrectly classified cases, highlighting the waveform regions that influenced model predictions. A more extensive demonstration, including 16 full-lead case studies with heatmaps, is available in Supplementary Section S8.

### 3.2 Performance benchmarking

ECG-XPLAIM outperformed both baselines across most tasks, while, compared to the pre-trained model, ECG-XPLAIM exhibited superior sensitivity but slightly lower specificity, suggesting a tendency to minimize false negatives (Table 3).

**Table 3:** Performance benchmarking of ECG-XPLAIM against baseline and external models.

Task	Sensitivity				Specificity				AUROC			
	ECG-XPLAIM	Vanilla CNN	GRU	External model**	ECG-XPLAIM	Vanilla CNN	GRU	External model	ECG-XPLAIM	Vanilla CNN	GRU	External model**
AFib	0.964 (0.944, 0.979)	0.644 (0.6, 0.686)*	0.966 (0.946, 0.98)	0.914 (0.886, 0.937)*	0.849 (0.835, 0.861)	0.837 (0.823, 0.85)*	0.83 (0.816, 0.844)*	0.919 (0.906, 0.931)*	0.958 (0.951, 0.965)	0.835 (0.823, 0.848)*	0.951 (0.943, 0.958)*	0.964 (0.956, 0.971)
STach	0.966 (0.946, 0.98)	0.892 (0.861, 0.918)*	0.954 (0.932, 0.971)	0.852 (0.818, 0.882)*	0.949 (0.941, 0.957)	0.935 (0.926, 0.944)*	0.894 (0.883, 0.905)*	0.98 (0.972, 0.985)*	0.983 (0.979, 0.988)	0.97 (0.965, 0.976)*	0.975 (0.97, 0.98)*	0.982 (0.976, 0.987)
RBBB	0.996 (0.986, 1)	0.986 (0.971, 0.994)	0.994 (0.983, 0.999)	0.922 (0.895, 0.944)*	0.936 (0.926, 0.944)	0.912 (0.901, 0.922)*	0.904 (0.892, 0.914)*	0.977 (0.969, 0.983)*	0.984 (0.98, 0.988)	0.975 (0.97, 0.98)*	0.98 (0.976, 0.985)	0.989 (0.985, 0.993)*
LBBB	0.988 (0.974, 0.996)	0.968 (0.949, 0.982)*	0.982 (0.966, 0.992)	0.93 (0.904, 0.951)*	0.894 (0.882, 0.905)	0.884 (0.872, 0.895)*	0.879 (0.866, 0.89)*	0.97 (0.962, 0.977)*	0.986 (0.983, 0.99)	0.979 (0.974, 0.984)*	0.985 (0.981, 0.989)*	0.985 (0.98, 0.99)
LAFB	0.694 (0.652, 0.734)	0.692 (0.649, 0.732)	0.692 (0.649, 0.732)	N/A	0.888 (0.876, 0.899)	0.862 (0.849, 0.874)*	0.883 (0.871, 0.894)	N/A	0.885 (0.874, 0.895)	0.882 (0.871, 0.892)	0.898 (0.888, 0.908)*	N/A
LQT	0.673 (0.577, 0.759)	0.7 (0.605, 0.784)	0.373 (0.282, 0.47)*	N/A	0.822 (0.809, 0.835)	0.612 (0.595, 0.629)*	0.841 (0.828, 0.853)*	N/A	0.81 (0.797, 0.823)	0.727 (0.713, 0.742)*	0.675 (0.66, 0.691)*	N/A
WPW	0.747 (0.633, 0.84)	0.347 (0.24, 0.465)*	0.187 (0.106, 0.293)*	N/A	0.901 (0.891, 0.911)	0.727 (0.711, 0.742)*	0.813 (0.799, 0.826)*	N/A	0.863 (0.852, 0.874)	0.562 (0.546, 0.579)*	0.521 (0.504, 0.537)*	N/A
PACE	0.959 (0.929, 0.978)	0.962 (0.933, 0.981)	0.969 (0.942, 0.986)	N/A	0.977 (0.972, 0.982)	0.825 (0.811, 0.838)*	0.682 (0.665, 0.698)*	N/A	0.985 (0.981, 0.989)	0.977 (0.972, 0.982)*	0.977 (0.972, 0.982)*	N/A

AFib, atrial fibrillation; AUROC, area under the receiver operating characteristic; CNN, convolutional neural network; GRU, gated recurrent unit; LAFB, left anterior fascicular block; LBBB, left bundle branch block; LQT, long QT; N/A, not available; PACE, paced rhythm; RBBB, right bundle branch block; STach, sinus tachycardia; WPW, Wolff-Parkinson-White pattern.

Metrics are reported with 95% confidence intervals; Values marked with an asterisk (*) indicate significant differences at 0.05 level, as compared to ECG-XPLAIM; ECG-XPLAIM was benchmarked against CNN and GRU across all eight classification labels, while the external model (27) was evaluated only on the four common labels supported by both models (AFib, STach, RBBB, LBBB); For each comparison, at least 500 records were sampled per label, except LQT (110), WPW (75) and PACE (290) due to limited data availability (more details in Supplementary Material).

In AFib detection, ECG-XPLAIM reached an AUROC of 0.958 (95% CI: 0.951–0.965), surpassing the vanilla CNN (0.835, 95% CI: 0.823–0.848) and GRU model (0.951, 95% CI: 0.943–0.958), while achieving comparable performance to the external model (0.964, 95% CI: 0.956–0.971). Sensitivity was 0.964 (95% CI: 0.944–0.979), significantly higher or comparable against all baselines, and specificity (0.849, 95% CI: 0.835–0.861) was higher than CNN and GRU, but slightly lower than the external model. For STach, ECG-XPLAIM achieved a sensitivity of 0.966 (95% CI: 0.946–0.980), a specificity of 0.949 (95% CI: 0.941–0.957), and an AUROC of 0.983 (95% CI: 0.979–0.988). Compared to the external model, ECG-XPLAIM reported higher sensitivity and slightly lower specificity, while AUROC was similar.

**Figure 5:**
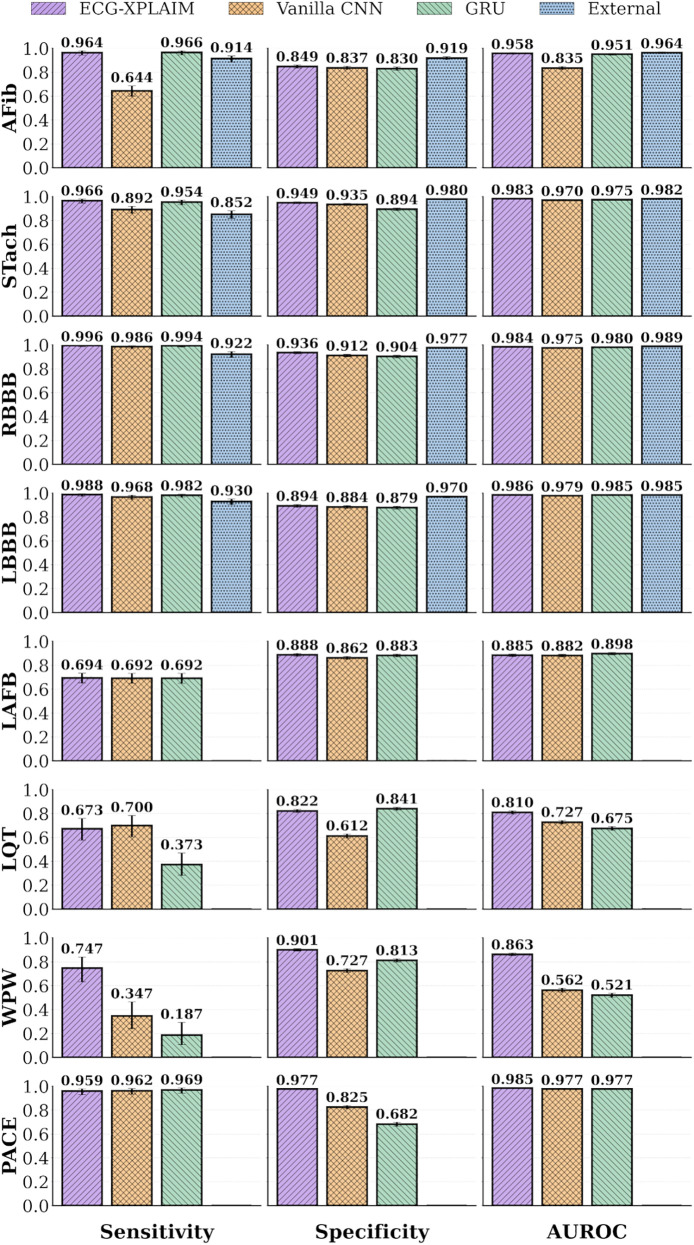
Performance bar graphs for specific arrhythmias. Classification performance metrics, including sensitivity, specificity, and area under the receiver operating characteristic (AUROC) curve, are displayed for individual arrhythmias. AFib, atrial fibrillation; CNN, convolutional neural network; External, external, pre-trained model (27); GRU, gated recurrent unit; LAFB, left anterior fascicular block; LBBB, left bundle branch block; LQT, long QT; PACE, paced rhythm; RBBB, right bundle branch block; STach, sinus tachycardia; WPW, Wolff-Parkinson-White pattern.

In conduction disturbances, ECG-XPLAIM maintained superior or comparable performance relative to the CNN and GRU baselines in most categories, with a slight exception in LAFB, where the GRU displayed a marginally higher AUROC. Sensitivity in detecting RBBB and LBBB was significantly higher than that of the external model (0.996, 95% CI: 0.986–1 for RBBB and 0.988, 95% CI: 0.974–0.996 for LBBB), although specificity was somewhat lower. For long QT (LQT) detection, ECG-XPLAIM achieved an AUROC of 0.81 (95% CI: 0.797–0.823), outperforming the CNN (0.727, 95% CI: 0.713–0.742) and GRU (0.675, 95% CI: 0.660–0.691). It also showed higher AUROC for WPW (0.863, 95% CI: 0.852–0.874) compared to CNN (0.562, 95% CI: 0.546–0.579) and GRU (0.521, 95% CI: 0.504–0.537), yielding also better sensitivity and specificity. For paced rhythm detection, ECG-XPLAIM surpassed both baselines in AUROC, with a value of 0.985 (95% CI: 0.981–0.989), while delivering a markedly higher specificity than both other models and comparable sensitivity. A summary of these comparative results is illustrated in Figure 5.

### 3.3 User experience and integration

To facilitate the adoption and practical utilization of ECG-XPLAIM, in both research and clinical environments, we provide pre-trained model weights and ready-to-use implementations for each classification task. We also provide the source code of model architecture, along with detailed documentation. A step-by-step user guide has been developed to assist clinicians and researchers in utilizing ECG-XPLAIM, outlining input formatting requirements, framework specifications, inference execution, and interpretation of outputs. Additionally, a dedicated Grad-CAM visualization module is included to support explainability assessment. This module enables users to generate heatmaps themselves that can point to ECG regions of importance, providing transparency into the model's decision-making process. By providing these tools and resources, we aim to position ECG-XPLAIM as a highly accessible, reproducible, and interpretable tool for AI-powered ECG analysis, both for clinical application and future research.

## 4 Discussion

### 4.1 Summary and interpretation

ECG-XPLAIM is a deep learning model that aims to balance high diagnostic accuracy with interpretability in automated ECG analysis. Its Inception-style architecture, optimized for time-series data, employs multi-scale processing with adaptive receptive fields, allowing the detection of short-duration waveform alterations, alongside global rhythm irregularities. Unlike traditional deep learning models that act as “black-box” classifiers, ECG-XPLAIM incorporates explainability mechanisms through one-dimensional Grad-CAM visualization, allowing for clinically meaningful interpretations and graspable explanations of its predictions.

Performance-wise, ECG-XPLAIM was assessed on held-out subsets of the development dataset (MIMIC-IV) for each task, where the model achieved metrics over 0.9 for all tasks. Most importantly, its diagnostic capability remained consistent on an external basis, when validated on the development-independent PTB-XL dataset. ECG-XPLAIM retained its competing performance, scoring metric values equal to or greater than 0.9 for most tasks, with only a few exceptions. Notably, certain arrhythmias, particularly LAFB, long QT, and WPW pattern, posed greater challenges in external evaluation, with sensitivity dropping to approximately 0.7–0.8, while specificity and AUROC remained consistently high. Additional analyses confirmed that introducing conventional signal preprocessing (bandpass 0.5–40 Hz, notch 50 Hz) did not materially improve performance compared to training on raw signals. In fact, pacing detection sensitivity declined under filtering, likely because sharp pacemaker spikes were attenuated. These findings (Supplementary Table S3) support training on raw signals for the primary claims, while providing reproducible code for transparency.

This study acknowledges that class imbalance, particularly for rare arrhythmias like WPW and LQT, remains a persistent challenge in automated ECG interpretation. Imbalances may result in reduced sensitivity for these categories and affect generalization to broader patient populations. Systematic reviews of data augmentation and synthetic signal generation techniques suggest that targeted strategies can ameliorate the impact of imbalance while enhancing the robustness and fairness of diagnostic models (31). To mitigate this, the model training employed a maximum cap of 50,000 samples per class where feasible, ensuring balanced representation across major arrhythmia categories. For WPW detection, due to data scarcity, a positive-to-negative sample ratio of approximately 1:12 (600 positive vs. 1,220 negative samples) was maintained, while LQT classification utilized around 39,000 samples per class. Other categories such as atrial fibrillation, sinus tachycardia, and conduction disturbances had similarly controlled sample sizes, with negative classes including non-target arrhythmic pathologies to reflect real-world complexity. These efforts aimed to limit dominant class bias without aggressive oversampling or augmentation. Supplementary analyses suggest that targeted augmentation and transfer learning strategies could further enhance detection of rare arrhythmias, supporting ongoing research in this direction (31).

The performance discrepancy between external and internal validation on specific labels, may stem from training set biases, class underrepresentation, or even inherent model limitations in capturing the subtle waveform characteristics. In particular, the reduced sensitivity for LQT (0.691) and WPW (0.773) in PTB-XL can be explained by cross-dataset domain shifts (differences in lead placement, sampling rates, acquisition chains, and annotation criteria), phenotype definition heterogeneity (e.g., QT correction formulas), and waveform ambiguity (e.g., when borderline QT prolongation or delta-like pre-excitation morphologies overlap with ectopy). These considerations highlight the importance of dataset-specific calibration and may motivate future domain adaptation strategies. Data augmentation (31–33), targeted fine-tuning (34), and signal pre-processing (35), might help enhance the detection of these patterns. Illustrative fine-tuning experiments on PTB-XL (0%–50% train/test splits) demonstrated that features transfer sufficiently across datasets: WPW AUROC improved steadily with additional PTB-XL data, LQT sensitivity spiked with very small fractions but at the expense of specificity, and TACHY/CD/PACE performance remained relatively robust. AUROC generally increased as more external data were used (Supplementary Table S5, Supplementary Figure S3). We emphasize that this violates strict external independence, so it is not part of our primary claims, but it highlights transfer learning as a promising future avenue.

Benchmarking against three counterpart models—a vanilla CNN, a more advanced two-layer GRU model, and an external, pre-trained ResNet-based architecture—revealed that ECG-XPLAIM generally demonstrates competitive performance. ECG-XPLAIM consistently outperformed both baseline CNN and GRU models, achieving higher sensitivity, specificity, and AUROC scores across most tasks. Compared to the external, pre-trained model, ECG-XPLAIM demonstrated higher sensitivity, leading to a slightly reduced specificity. This preference for minimizing false negatives aligns with its potential role as a screening tool, where missing critical arrhythmias is more concerning than erroneously flagging some normal cases. Benchmarking against such leading architectures demonstrates the competitive diagnostic performance of the present approach. Importantly, comparison with large-scale neural network frameworks for ECG interpretation, such as that developed by Ribeiro et al., highlights the added clinical value of integrated interpretability, which is increasingly recognized as a prerequisite for real-world deployment in cardiology settings (27).

### 4.2 Clinical applicability and added value

ECG-XPLAIM is designed to integrate seamlessly into clinical workflows by prioritizing both diagnostic accuracy and interpretability. Its high sensitivity ensures that clinically significant arrhythmias are detected early, aiding in timely referrals to cardiology specialists and reducing the risk of underdiagnosis in severe conditions such as conduction blocks or arrhythmias predisposing to ventricular events. Additionally, it enhances efficiency in high-throughput diagnostic environments by assisting in automated triaging of abnormal ECGs, reducing the burden on specialists, offering a fatigue-free screening solution, and facilitating early identification of high-risk patients.

Unlike many prior approaches that primarily distinguish normal from abnormal ECGs, ECG-XPLAIM focuses on challenging arrhythmias and leverages multi-class, overlap-tolerant training. Negative classes in each task are not purely “normal” recordings but instead exclude only the target arrhythmic entity while potentially containing other conditions. Consequently, ECG-XPLAIM learns to differentiate subtle, overlapping abnormalities—a skill crucial in real-world practice where arrhythmias often coexist or mimic one another. Furthermore, ECG-XPLAIM was specifically trained on well-defined classification tasks, ensuring a balanced representation across involved classes and focusing only on arrhythmias that are challenging to differentiate. This task-specific design increases its applicability in real-world settings, where ECGs often present overlapping abnormalities that require fine-grained discrimination.

ECG-XPLAIM represents a highly scalable solution, capable of handling large-scale ECG datasets, supporting both high-volume batch processing and low-latency real-time inference, making it suitable for both retrospective research and live clinical deployments. From a technical perspective, it exhibits short inference times (4.5–16 milliseconds per 10-s ECG), enabling near real-time deployment in edge- or server-based infrastructures. The model is open-source, enabling research groups to extend its architecture, adapt it for novel classification tasks, and implement custom modifications tailored to specific clinical needs. The availability of pre-trained weights facilitates direct deployment without extensive retraining, while also allowing for transfer learning and fine-tuning on new datasets, significantly reducing computational costs and making it accessible to a broader user base.

An additional threshold analysis (Supplementary Table S6) demonstrated how sensitivity and specificity trade-offs evolve across thresholds 0.0–1.0. For example, TACHY achieved a balanced operating point at 0.4–0.5 (SEN 0.968–0.955, SPE 0.952–0.969), CD balanced at 0.5, and PACE maintained excellent performance across a wide range of thresholds. In contrast, LQT sensitivity dropped steeply as thresholds increased, suggesting that lower thresholds (≤0.3–0.4) may be preferable in screening contexts, but not for disease confirmation, due to high risk for false positives. These findings support the potential for site-specific threshold personalization, where operating points can be pre-specified depending on whether the model is used for broad screening (favoring sensitivity) or confirmatory diagnostics (favoring specificity).

More broadly, ECG-XPLAIM fits into the growing role of AI in electrophysiology (EP) workflows, which extends beyond ECG classification into procedural guidance, ablation planning, and arrhythmia risk stratification (36). AI tools are increasingly applied for automated mapping of atrial and ventricular arrhythmias, predicting catheter ablation outcomes, optimizing device programming, and guiding individualized risk assessment for sudden cardiac death. Within this framework, ECG-based algorithms such as ours provide the critical front-end signal interpretation layer: by ensuring reliable, explainable detection of arrhythmias and conduction disturbances, ECG-XPLAIM can serve as the entry point that feeds into downstream EP workflows, including rhythm monitoring, decision-support in invasive procedures, and integration with longitudinal risk prediction models. In this way, ECG-XPLAIM's emphasis on transparency and adaptability positions it as a foundational component in the translational pipeline of AI in EP. Explainable and open-source models such as ours align with these translational goals, as highlighted in recent consensus perspectives on AI in EP (13).

### 4.3 Explainability and trust in AI-driven diagnostics

A primary barrier to the clinical adoption of deep learning in healthcare is the “black-box” nature of most models. The interpretability of deep learning models applied to electrocardiogram analysis is increasingly acknowledged as essential for facilitating clinical adoption and patient safety. Contemporary literature highlights that XAI methods require rigorous evaluation to ensure reliability and relevance in clinical practice. Salih et al. conducted a systematic review of XAI evaluations in cardiology, revealing that only a minority of studies applied systematic assessment: 37% benchmarked XAI quality based on prior literature, 11% involved clinicians as domain experts, and 11% relied on quantitative proxies or statistical analysis, while 43% did not assess explanation quality at all. The authors advocate for formal, multi-dimensional frameworks that include faithfulness, fidelity, and direct clinician feedback, emphasizing that thorough evaluation of explanations is critical for the development of trustworthy and safe AI models in medicine (14).

Recent advances also underscore the utility of combining multiple interpretability techniques and actively involving clinicians in validation processes. Zhang et al. demonstrated the application of Grad-CAM in medical text classification, illustrating how visualization of salient features through heatmaps can intuitively communicate the basis for predictions to human users. Their comparative study using word embeddings and various classifier architectures (Word2Vec, BERT, ResNet, CNN, Bi-LSTM) showed that integrating Grad-CAM with high-performing deep learning models enables more transparent identification of decision-influencing input signals. The study found that Grad-CAM visualization reliably highlighted text regions most relevant to the model's outputs, supporting the practical integration of XAI in clinical decision making and error analysis (37).

ECG-XPLAIM confronts this challenge by integrating Grad-CAM-based explainability, enabling visualization of the waveform regions that contribute most to its predictions. This transparency fulfills several objectives. First, it strengthens clinician trust by revealing the model's decision-making process. Second, it facilitates potential feature discovery, unearthing subtle waveform variations that may carry clinical significance. Third, it supports adherence to emerging regulatory guidelines—such as those from the U.S. Food and Drug Administration (FDA) and the European Medicines Agency (EMA)—that increasingly emphasize interpretability requirements for medical AI systems (38, 39). Finally, it assists in error analysis: highlighting waveforms that led to misclassifications allows targeted improvements to the model's training and architecture.

In this study, Grad-CAM visualizations helped pinpoint areas of interest, in both correctly identified and misclassified cases. For AFib, the model consistently focused on the absence of P waves in the pre-QRS region (Figure 4A), while the STach detection was primarily driven by P-wave presence and regularly appearing points of interest that signify rhythmicity (Figure 4B). RBBB and LBBB cases showed strong attention to the QRS complex morphology (Figures 4D,E), while in LAFB, the model seemed to capture the associated axis deviation-related changes (Figure 4F). For LQT, ECG-XPLAIM correctly identified the QT interval by focusing on the onset and termination of the repolarization phase in certain beats (Figure 4C). WPW classification relied on the characteristic delta wave and the PR interval (Figure 4G). Paced rhythm cases were accurately identified by highlighting both atrial and ventricular pacing spikes across all beats (Figure 4H).

On the other hand, false classifications revealed cases where the model's attention was misdirected, particularly for arrhythmias with lower sensitivities. In an AFib false positive case, ECG-XPLAIM incorrectly interpreted a conduction delay due to first-degree AV block (I-AVB) as an absent P wave (Figure 4I), demonstrating a potential bias in P-wave localization. Conversely, in a false negative STach case, the model correctly detected rapid rhythm but misclassified it as AFib due to near-fusion of the P wave with the preceding T wave at high heart rates. Bundle branch block misclassifications were primarily linked to variations in QRS duration that seemed borderline. False positive classifications often involved misinterpretation of extrasystoles or morphological changes in the QRS complex (Figure 4M). For LQT false negatives, the model's attention was restricted to a segment within the repolarization phase rather than spanning the full QT interval, for some cases (Figure 4J). In WPW false positive examples, ECG-XPLAIM placed significant focus on the pre-QRS regions of wide-QRS extrasystoles, mistaking premature beats for delta waves, indicating a bias in distinguishing abnormal conduction patterns. Similarly, a false positive classification of pacing occurred when the model misinterpreted a narrow QRS complex as a pacing spike (Figure 4L). (Detailed examples with explanations are offered in Supplementary Section S8.) These findings indicate the origin of prediction faults and suggest strategies to mitigate them, such as augmenting training data with borderline and atypical presentations (31).

Despite its utility, Grad-CAM is not a perfect solution. Although it highlights influential waveform regions, it does not fully elucidate the underlying rationale—why certain features are attributed to one arrhythmia rather than another (37). Future research could explore more advanced or complementary explainable AI techniques, potentially integrating rule-based logic or interpretability frameworks that capture inter-lead relationships. These refinements may further reduce model rigidity and enhance its ability to handle the complexities of real-world ECG data.

### 4.4 Limitations and future directions

Despite strong performance and explainability, ECG-XPLAIM faces certain limitations. Class imbalance was present across tasks, particularly for rare arrhythmias such as WPW and LQT, where positive samples were substantially fewer than negatives (e.g., WPW 600 vs. 1,220). Although we capped maximum samples per class and applied balanced mini-batching, residual imbalance may have contributed to lower external sensitivities. Future work could leverage more targeted data collection or dedicated augmentation strategies for ECG signals to enrich rare classes and improve model calibration (31). Importantly, the reliability of diagnostic labels was confirmed via inter-observer agreement analysis: in a random 10% subset of MIMIC-IV, kappa values ranged from 0.990 to 0.998 across all labels, supporting the sufficiency of pre-annotations (Supplementary Table S4). Finally, real-world clinical performance can only be validated prospectively; although robust, retrospective testing on MIMIC-IV and PTB-XL does not guarantee identical outcomes in diverse clinical environments.

While Grad-CAM visualizations partly address the interpretability gap, they do not provide an explicit rationale for how certain features lead to a diagnosis. For instance, identifying a lengthened QRS complex does not clarify how the model distinguishes between RBBB and LBBB. More sophisticated XAI methods could further demystify the decision process and illuminate nuanced inter-lead relationships that underlie arrhythmia detection.

Finally, exploratory transfer learning experiments on PTB-XL (Section 6 of the Supplementary Material) demonstrated that ECG-XPLAIM's feature representations are transferable across datasets, particularly for WPW and LQT detection. While these experiments can improve metrics, such as AUROC and sensitivity, for specific labels under certain splits, they are illustrative only, as they break the requirement for independence in external validation. Nonetheless, they motivate future research directions.

Future research will focus on refining ECG-XPLAIM's generalization and interpretability. Fine-tuning on localized, hospital-specific datasets could account for regional ECG variations and acquisition protocols, while federated learning approaches may broaden the model's adaptability without centralized data pooling (40). Investigating additional XAI techniques or combining Grad-CAM with rule-based logic could strengthen interpretability and expedite regulatory acceptance. Finally, prospective clinical trials will be essential to evaluate real-world feasibility, confirm performance in diverse patient populations, and measure clinical outcomes and workflow improvements attributable to ECG-XPLAIM's integration.

## 5 Conclusions

In this work, we propose ECG-XPLAIM, an explainable deep learning model for automated arrhythmia detection, which demonstrates robust generalization in both the MIMIC-IV and PTB-XL datasets. ECG-XPLAIM outperforms baseline CNN and advanced GRU models in most classification tasks and offers performance comparable to a state-of-the-art pre-trained network, with a priority to minimize the risk of underdiagnosis. By emphasizing sensitivity, it reduces missed diagnoses, making it particularly well-suited for screening workflows. Its integrated Grad-CAM mechanism provides interpretable visualizations of the waveform regions guiding classification, simultaneously furnishing critical feedback for model refinement. Additional analyses confirmed the robustness of training on raw signals, and the flexibility to adapt performance through threshold calibration or transfer learning to new datasets. These features strengthen the model's translational potential. Although further optimization for rare arrhythmias, larger datasets, and real-world prospective validation are warranted, ECG-XPLAIM's scalability, open-source implementation, and rapid inference, position it as a valuable tool for integrating AI-driven cardiac diagnostics into clinical practice.

## Data Availability

The datasets presented in this study can be found in online repositories. The names of the repository/repositories and accession number(s) can be found below: https://physionet.org/. The ECG-XPLAIM source code, along with use instructions, are available on GitHub at https://github.com/ppantele/ECG-XPLAIM. The trained weights of ECG-XPLAIM for each task are available on Zenodo at https://zenodo.org/records/14968732.
