# Supplementary material for: ECG-XPLAIM: eXPlainable Locally-adaptive Artificial Intelligence Model for arrhythmia detection from large-scale electrocardiogram data
Source: Front Cardiovasc Med. 2025 Oct 16;12:1659971. doi: 10.3389/fcvm.2025.1659971 (PMC12573295; doi:10.3389/fcvm.2025.1659971)
Supplement: Supplementary file 1 [file Datasheet1.pdf]

Supplementary Material

1 Label Extraction Strategy

The labels for electrocardiogram (ECG) classification were extracted using predefined search terms from diagnostic reports in both the *MIMIC-IV (ECG)* and *PTB-XL* datasets (available at PhysioNet). For *MIMIC-IV*, labels were identified by searching concatenated fields (‘report_0’ to ‘report_17’) within the ‘machine_measurements.csv’ file. In *PTB-XL*, labels were derived from the ‘scp_codes’ field in the ‘ptbxl_database.csv’ file. The final keyword mappings used for automated label extraction are summarized below:

Supplementary Table 1. Label extraction search terms for MIMIC-IV and PTB-XL datasets.

Arrhythmia	*MIMIC-IV dataset*	*PTB-XL dataset*
Atrial fibrillation (AFib)	‘afib’, ‘atrial fib’	‘AFIB’
Right bundle branch block (RBBB)	‘rbbb’, ‘right b’	‘CRBBB’
Left bundle branch block (LBBB)	‘lbbb’, ‘left b’	‘CLBBB’
Left anterior fascicular block (LAFB)	‘lafb’, ‘left ant’	‘LAFB’
Sinus tachycardia (STach)	‘sinus tach’	‘STACH’
Long QT pattern (LQT)	‘long qt’, ‘longed qt’	‘LNGQT’
Wolff-Parkinson-White (WPW)	‘wpw’, ‘wolf’	‘WPW’
Paced rhythm (PACE)	‘paci’, ‘pace’	‘PACE’

To ensure labeling accuracy, a randomly selected 10% of *MIMIC-IV* was manually validated by expert clinicians, for each task-specific subset used during training and internal evaluation. Similarly, the complete *PTB-XL* subsets used for external evaluation and benchmarking, were manually verified.

2 Deep Learning Model Architectures

This section provides details on the architecture of the models used in this study. All model architectures were implemented using TensorFlow/Keras in Python 3.

2.1 ECG-XPLAIM model

A custom Inception-style convolutional neural network (CNN) optimized for time-series data and ECG analysis, featuring three residual blocks, each with two Inception modules incorporating parallel convolutional filters (in this case, with lengths: 2, 10, 40). Code and trained weights are available on GitHub (https://github.com/ppantele/ECG-XPLAIM) and Zenodo (https://zenodo.org/records/14968732).

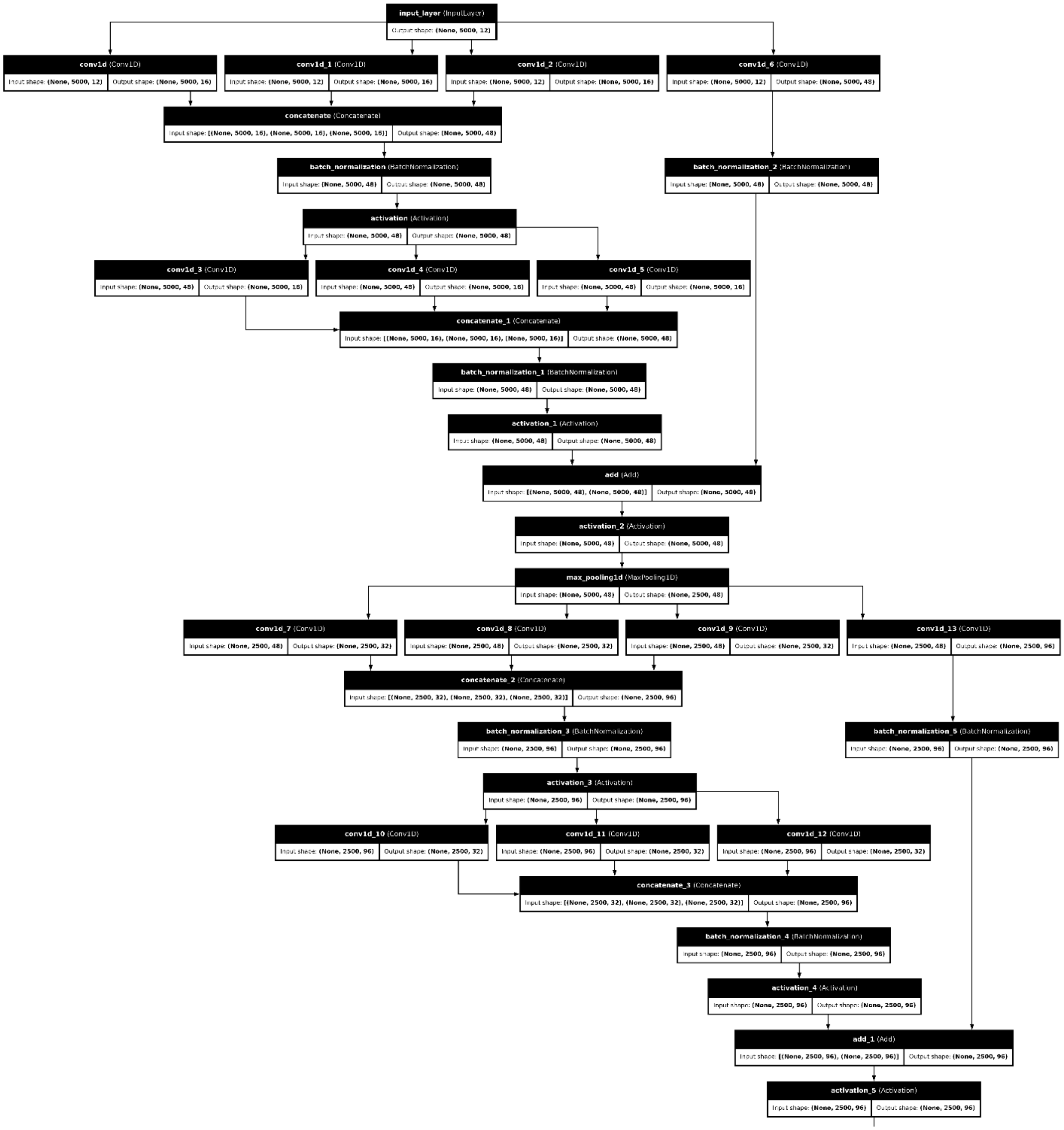

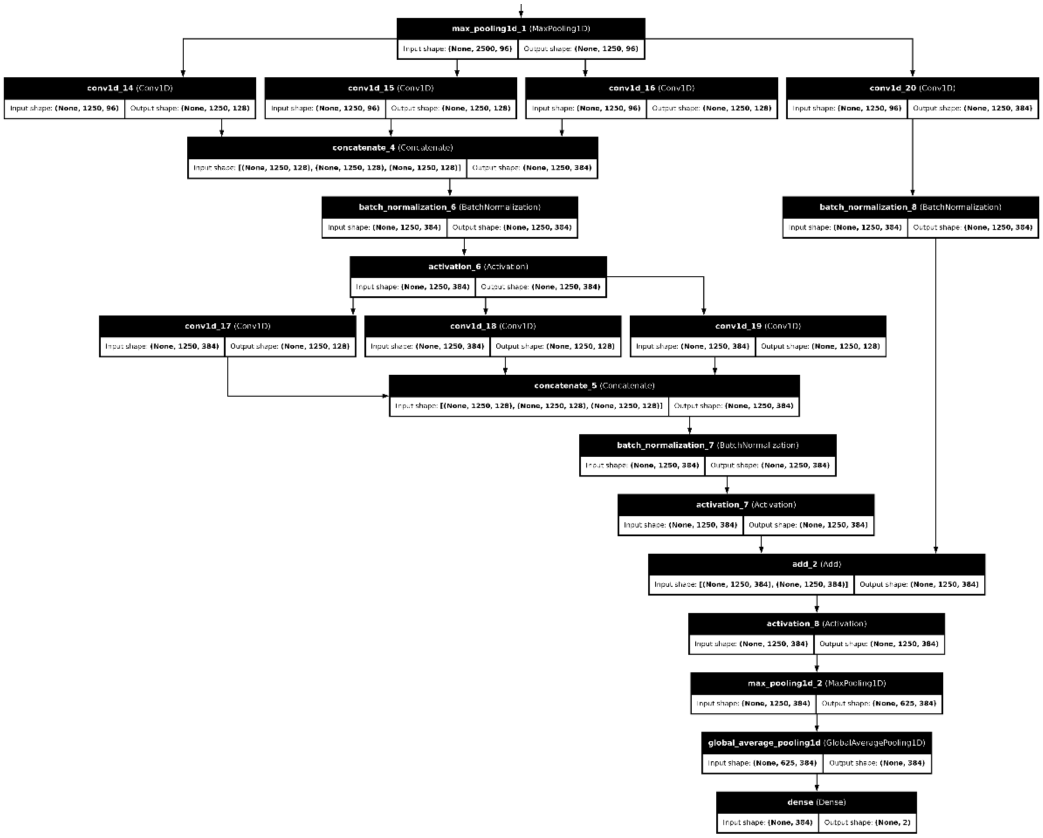

Supplementary Figure 1. Architecture of ECG-XPLAIM.

2.2 Baseline models (vanilla CNN and advanced GRU)

A simple CNN with three one-dimensional convolutional layers, each followed by batch normalization and max pooling, was used to serve as a baseline for performance comparison. Additionally, a recurrent model designed for sequential ECG processing, consisting of two stacked gated recurrent unit (GRU) layers followed by a dense classification module, was also employed.

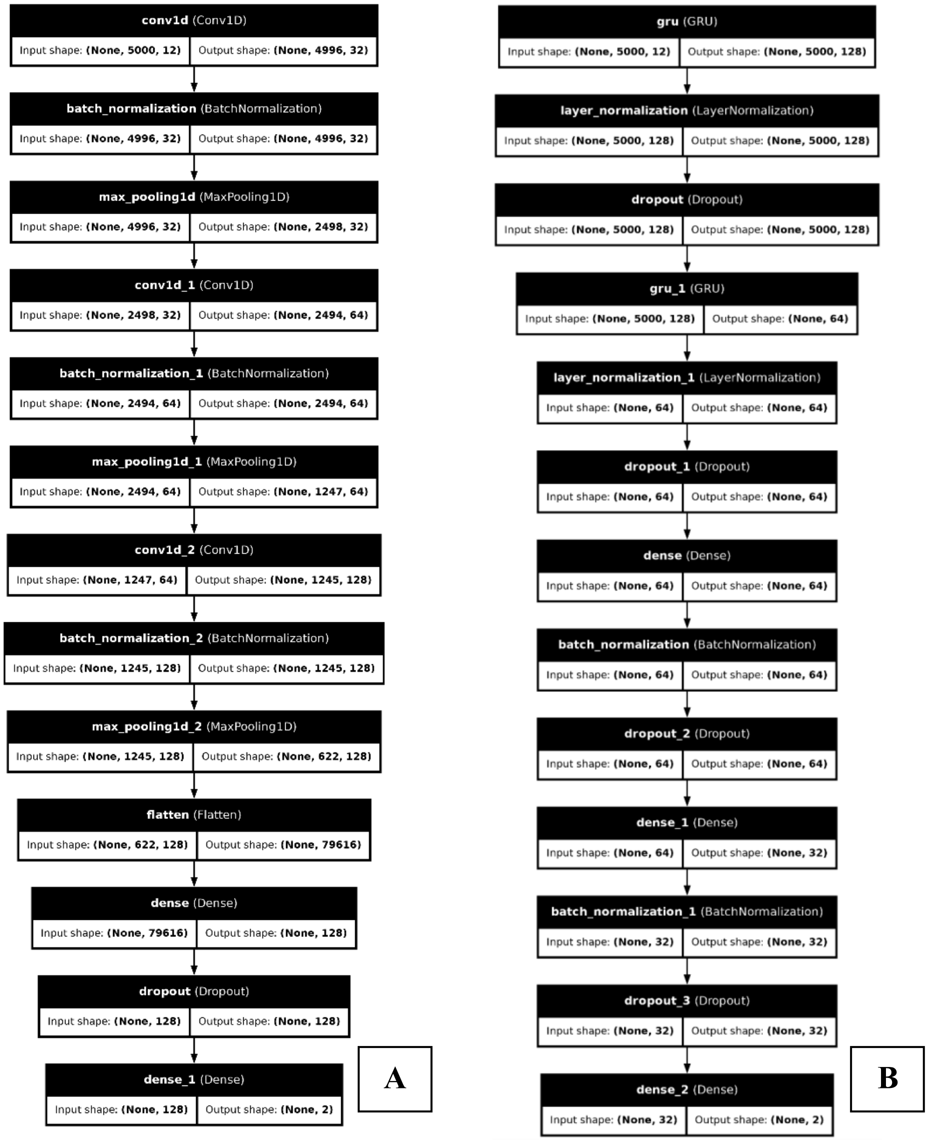

Supplementary Figure 2. Architectures of baselines, vanilla CNN (A), and advanced GRU (B).

2.3 External model details and adaptation

A pre-trained deep ResNet model for 12-lead ECG classification. The setup was adapted to match the model's specifications, including data downsampling to 400Hz, sample padding to 4,096 data points, and lead configuration adjustment. The model is available online (Ribeiro AH et al, 2020, doi: 10.1038/s41467-020-15432-4).

3 Training and Validation Strategy

The classification tasks were selected to represent clinically relevant ECG differentiation scenarios. The five tasks included distinguishing between atrial fibrillation (AFib) and sinus tachycardia (STach), various conduction disturbances (RBBB, LBBB, LAFB), as well as identifying long QT (LQT), Wolff-Parkinson-White pattern (WPW), and paced rhythms (PACE).

For each classification task, the *MIMIC-IV* dataset was split into development (dev) and test sets, with the dev set further divided into training and validation subsets. The training subset was used to train task-specific models, which were constantly monitored on the validation subset. The independent test set was reserved exclusively for internal evaluation. For external evaluation and benchmarking, independent sets were randomly sampled from the *PTB-XL* dataset, following the same task-specific strategy.

To mitigate class imbalance, particularly for rare conditions, all sets were label-wise balanced both at the global level and within individual batches (for development subsets). The class balance was maintained within each batch, to minimize the risk of early gradient drift caused by successive feeding of unbalanced batches and to stabilize the learning procedure.

For each classification task, the negative class included ECGs that excluded only the target arrhythmia while allowing other, coexisting abnormalities. This design, not restricting the negative class only to normal/healthy samples, ensured that the model learned to differentiate between overlapping ECG patterns, reflecting real-world diagnostic complexity.

Supplementary Table 2. Dataset splits for training, validation, and testing with corresponding sample sizes.

Task	*MIMIC-IV (n=800,035)*					*PTB-XL (n=21,799)*	
	*Available number of samples**	*Dev set – Sample size*	*Batch size*	*Train / validation number of batches*	*Internal evaluation test set – Sample size*	*Available number of samples**	*External evaluation test set – Sample size*
AFib vs. STach vs. Neg	81,330 vs. 69,260	50,000 each	128	1130 / 10	1,000 each	1,514 vs 826	1,500 vs. 800 vs. 1,500
RBBB vs. LBBB vs. LAFB vs. Neg	65,924 vs. 29,694 vs. 48,341	25,000 each	128	740 / 10	1,000 each	541 vs 536 vs 1,623	500 vs. 500 vs. 1,600 vs. 1,600

LQT vs. Neg	39,533	39,000 each	128	580 / 10	1,000 each	117	110 each
WPW vs. Neg	616	610 vs. 1220**	32**	45 / 5	100 each	79	75 each
PACE vs. Neg	31,750	30,000 each	128	440 / 10	1,000 each	294	290 each

* The number of negative samples is not explicitly listed (equal to the total dataset size minus the sum of positive labels); ** A 1:2 positive-to-negative ratio, with a smaller batch size (32), was applied for WPW due to the limited number of positive samples; AFib, Atrial fibrillation; STach, Sinus tachycardia; Neg, Negative class (excluding only the target arrhythmias); RBBB, Right bundle branch block; LBBB, Left bundle branch block; LAFB, Left anterior fascicular block; LQT, Long QT; WPW, Wolff-Parkinson-White pattern; PACE, Paced rhythm; Dev, Development set (training and validation combined).

4 Effect of Pre-processing on Model Performance

To assess the influence of signal pre-processing on diagnostic performance, we trained and evaluated additional ECG-XPLAIM variants using two widely applied filtering strategies:

- Bandpass filtering (0.5–40 Hz, 4th-order zero-phase Butterworth): typically applied in clinical practice to suppress baseline wander and high-frequency noise, but potentially attenuating sharp patterns and fast components.
- Notch filtering (50 Hz, quality factor Q=30): used to suppress powerline interference while preserving most of the physiological frequency spectrum.

These were compared against the baseline training setup directly on raw signals. The following table demonstrates the results.

Supplementary Table 3. Effect of signal pre-processing (bandpass and notch filtering) on external evaluation performance (PTB-XL).

Task	*No pre-processing*			*Bandpass filter*			*Notch filter*		
	SEN	*SPE*	*AUC*	*SEN*	*SPE*	*AUC*	*SEN*	*SPE*	*AUC*
TACHY									
AFib	0.954 (0.942, 0.964)	0.964 (0.955, 0.971)	0.988 (0.984, 0.991)	0.963 (0.953, 0.972)	0.929 (0.917, 0.939)	0.985 (0.981, 0.989)	0.92 (0.905, 0.933)	0.963 (0.955, 0.971)	0.985 (0.981, 0.989)
STach	0.956 (0.94, 0.969)	0.974 (0.967, 0.979)	0.991 (0.988, 0.994)	0.924 (0.903, 0.941)	0.988 (0.983, 0.991)	0.992 (0.989, 0.995)	0.927 (0.907, 0.944)	0.99 (0.986, 0.993)	0.992 (0.989, 0.995)
macro-avg	0.955	0.969	0.99	0.944	0.958	0.988	0.924	0.976	0.988
CD									
RBBB	0.996 (0.986, 1)	0.966 (0.959, 0.972)	0.994 (0.992, 0.997)	0.99 (0.977, 0.997)	0.951 (0.944, 0.958)	0.993 (0.991, 0.996)	0.992 (0.98, 0.998)	0.961 (0.954, 0.967)	0.993 (0.991, 0.996)
LBBB	0.99 (0.977, 0.997)	0.927 (0.918, 0.936)	0.99 (0.987, 0.993)	0.972 (0.953, 0.985)	0.959 (0.952, 0.966)	0.991 (0.988, 0.994)	0.986 (0.971, 0.994)	0.917 (0.908, 0.926)	0.991 (0.988, 0.994)
LAFB	0.714 (0.691, 0.736)	0.962 (0.954, 0.97)	0.946 (0.939, 0.953)	0.738 (0.717, 0.757)	0.955 (0.946, 0.963)	0.94 (0.935, 0.946)	0.651 (0.627, 0.675)	0.953 (0.944, 0.961)	0.943 (0.935, 0.95)
macro-avg	0.9	0.952	0.977	0.9	0.955	0.975	0.876	0.944	0.976

LQT	0.691 (0.596, 0.776)	0.864 (0.785, 0.922)	0.878 (0.835, 0.922)	0.6 (0.502, 0.692)	0.936 (0.873, 0.974)	0.872 (0.83, 0.915)	0.618 (0.521, 0.709)	0.848 (0.789, 0.906)	0.878 (0.835, 0.921)
WPW	0.773 (0.662, 0.862)	0.973 (0.907, 0.997)	0.895 (0.846, 0.944)	0.787 (0.677, 0.873)	0.893 (0.801, 0.953)	0.90 (0.854, 0.946)	0.76 (0.647, 0.851)	0.96 (0.888, 0.992)	0.89 (0.852, 0.928)
PACE	0.96 (0.928, 0.981)	0.988 (0.965, 0.998)	0.993 (0.985, 1)	0.893 (0.852, 0.926)	0.952 (0.92, 0.973)	0.968 (0.953, 0.982)	0.945 (0.912, 0.968)	0.983 (0.96, 0.994)	0.99 (0.982, 0.998)

Metrics are reported with 95% confidence intervals; Abbreviations: AFib, Atrial fibrillation; AUC, Area under the receiver operating characteristic curve; CD, Conduction disturbance task; LAFB, Left anterior fascicular block; LBBB, Left bundle branch block; LQT, Long QT; Macro-avg, Macro-averaged metric; PACE, Paced rhythm; RBBB, Right bundle branch block; SEN, Sensitivity; SPE, Specificity; STach, Sinus tachycardia; TACHY, Tachycardia task; WPW, Wolff-Parkinson-White pattern.

5 Inter-observer Agreement Metrics

To evaluate the reliability of diagnostic labels used for training and validation, we assessed inter-observer agreement between pre-annotated labels (derived from database reports) and manual expert annotation. A randomly selected 10% subset of the MIMIC-IV dataset (n: 80,003 ECGs) was independently reviewed by clinicians. Agreement was quantified using Cohen's κ (kappa), with 1,000 bootstrap resamples to estimate 95% confidence intervals (CIs) for each label. Agreement was consistently high across most arrhythmia categories, supporting the validity of the pre-annotated labels for large-scale model development. Kappa values exceeded 0.99 across all tasks, indicating almost perfect agreement.

Supplementary Table 4. Inter-observer agreement between database-derived labels and manual expert annotation on 10% of MIMIC-IV (n: 80,003).

Label	*Cohen's kappa (95% CIs)*
AFib	0.997 (0.996, 0.998)
STach	0.996 (0.995, 0.997)
RBBB	0.998 (0.997, 0.998)
LBBB	0.991 (0.988, 0.993)
LAFB	0.997 (0.996, 0.998)
LQT	0.994 (0.992, 0.996)
WPW	0.990 (0.981, 0.998)
PACE	0.993 (0.990, 0.995)

Cohen's kappa values are reported with 95% confidence intervals (1,000 bootstrap resamples); Abbreviations: AFib, Atrial fibrillation; LAFB, Left anterior fascicular block; LBBB, Left bundle branch block; LQT, Long QT; PACE, Paced rhythm; RBBB, Right bundle branch block; STach, Sinus tachycardia; WPW, Wolff-Parkinson-White pattern.

6 Transfer Learning on PTB-XL: Fine-tuning with Different Train/Test Splits

This section reports the results of transfer learning experiments, where the pretrained ECG-XPLAIM model (trained on MIMIC-IV) was fine-tuned on PTB-XL using varying proportions of training and test data (0/100, 5/95, 10/90, 20/80, and 50/50). These experiments were conducted to explore the potential for feature transferability and domain adaptation across datasets.

Key findings:

- Tachyarrhythmias (AFib, STach): Metrics remained consistently high across splits. Small fractions of PTB-XL data (5–10%) modestly improved sensitivity for AFib, while specificity remained stable. STach showed variability, with sensitivity dips at lower fractions (5–20%) but recovery at 50%.
- Conduction disturbances: RBBB performance decreased at small fine-tuning fractions but recovered with larger splits. LBBB showed strong specificity at 5% fine-tuning (1.00), but at the cost of lower sensitivity, stabilizing with ≥20%. LAFB demonstrated the most benefit from fine-tuning, with sensitivity markedly improving at 5–20% compared to the baseline.
- Rare conditions (LQT, WPW): Performance fluctuated substantially. For LQT, fine-tuning with small fractions caused sensitivity to spike (0.98–0.99) but at the expense of specificity (<0.40), reflecting overfitting to few positive cases. Larger fractions (20–50%) restored balanced metrics. WPW exhibited similar trends: modest improvements in sensitivity at the cost of specificity, yet converging at higher AUROC at larger splits.
- Paced rhythms: Performance was similar across all splits, with minimal change in sensitivity, specificity, and AUROC.

Overall, fine-tuning on PTB-XL confirmed that ECG-XPLAIM's learned representations can transfer across datasets, but highlighted the risk of overfitting. These results should be considered illustrative, as they violate strict independence assumptions and are not part of the primary claims.

Supplementary Table 5. Transfer learning experiments: performance of ECG-XPLAIM after fine-tuning on PTB-XL with different train/test splits.

Task	*0/100%*			*5/95%*			*10/90%*			*20/80%*			*50/50%*		
	SEN	*SPE*	*AUC*	*SEN*	*SPE*	*AUC*	*SEN*	*SPE*	*AUC*	*SEN*	*SPE*	*AUC*	*SEN*	*SPE*	*AUC*
TACHY															
AFib	0.954 (0.942, 0.964)	0.964 (0.955, 0.971)	0.988 (0.984, 0.991)	0.961 (0.949, 0.971)	0.959 (0.949, 0.968)	0.99 (0.986, 0.994)	0.965 (0.953, 0.974)	0.953 (0.943, 0.961)	0.988 (0.985, 0.992)	0.969 (0.959, 0.978)	0.952 (0.942, 0.961)	0.987 (0.983, 0.99)	0.989 (0.979, 0.998)	0.973 (0.963, 0.982)	0.99 (0.986, 0.993)
STach	0.956 (0.94, 0.969)	0.974 (0.967, 0.979)	0.991 (0.988, 0.994)	0.909 (0.883, 0.93)	0.985 (0.979, 0.989)	0.989 (0.986, 0.993)	0.926 (0.905, 0.944)	0.991 (0.986, 0.994)	0.993 (0.99, 0.996)	0.907 (0.884, 0.927)	0.987 (0.982, 0.991)	0.989 (0.986, 0.992)	0.957 (0.934, 0.977)	0.989 (0.984, 0.993)	0.99 (0.986, 0.992)
macro-avg	0.955	0.969	0.99	0.935	0.972	0.99	0.946	0.972	0.99	0.938	0.97	0.988	0.973	0.981	0.99

CD															
RBBB	0.996 (0.986, 1)	0.966 (0.959, 0.972)	0.994 (0.992, 0.997)	0.919 (0.891, 0.942)	0.991 (0.987, 0.994)	0.99 (0.987, 0.993)	0.851 (0.816, 0.882)	0.991 (0.987, 0.994)	0.994 (0.991, 0.996)	0.944 (0.918, 0.964)	0.987 (0.982, 0.99)	0.995 (0.993, 0.998)	0.969 (0.939, 0.986)	0.981 (0.973, 0.987)	0.994 (0.99, 0.997)
LBBB	0.99 (0.977, 0.997)	0.927 (0.918, 0.936)	0.99 (0.987, 0.993)	0.607 (0.561, 0.65)	1.0 (0.999, 1.0)	0.97 (0.965, 0.976)	0.885 (0.852, 0.913)	0.992 (0.989, 0.995)	0.989 (0.985, 0.992)	0.915 (0.883, 0.94)	0.994 (0.991, 0.997)	0.992 (0.989, 0.995)	0.909 (0.865, 0.942)	0.993 (0.988, 0.997)	0.989 (0.984, 0.994)
LAFB	0.714 (0.691, 0.736)	0.962 (0.954, 0.97)	0.946 (0.939, 0.953)	0.979 (0.971, 0.986)	0.834 (0.818, 0.849)	0.964 (0.958, 0.97)	0.964 (0.953, 0.973)	0.911 (0.898, 0.922)	0.979 (0.974, 0.984)	0.955 (0.942, 0.966)	0.925 (0.913, 0.936)	0.98 (0.975, 0.985)	0.91 (0.888, 0.929)	0.945 (0.93, 0.957)	0.978 (0.971, 0.984)
macro-avg	0.9	0.952	0.977	0.835	0.942	0.975	0.9	0.965	0.987	0.938	0.969	0.989	0.929	0.973	0.987
LQT	0.691 (0.596, 0.776)	0.864 (0.785, 0.922)	0.878 (0.835, 0.922)	0.981 (0.932, 0.998)	0.226 (0.151, 0.318)	0.824 (0.772, 0.875)	0.99 (0.945, 1.0)	0.313 (0.224, 0.414)	0.806 (0.75, 0.861)	0.866 (0.773, 0.931)	0.745 (0.644, 0.829)	0.906 (0.863, 0.949)	0.942 (0.841, 0.988)	0.569 (0.432, 0.698)	0.917 (0.866, 0.969)
WPW	0.773 (0.662, 0.862)	0.973 (0.907, 0.997)	0.895 (0.846, 0.944)	0.764 (0.649, 0.856)	0.971 (0.901, 0.997)	0.908 (0.86, 0.955)	0.75 (0.626, 0.85)	0.958 (0.881, 0.991)	0.929 (0.885, 0.972)	0.77 (0.645, 0.868)	0.915 (0.813, 0.972)	0.91 (0.859, 0.961)	0.757 (0.588, 0.882)	0.921 (0.786, 0.983)	0.913 (0.849, 0.977)
PACE	0.96 (0.928, 0.981)	0.988 (0.965, 0.998)	0.993 (0.985, 1)	0.96 (0.929, 0.98)	0.982 (0.958, 0.994)	0.992 (0.984, 0.999)	0.949 (0.915, 0.973)	0.992 (0.973, 0.999)	0.989 (0.98, 0.998)	0.97 (0.939, 0.988)	0.983 (0.956, 0.995)	0.995 (0.988, 1)	0.924 (0.867, 0.961)	0.973 (0.931, 0.992)	0.981 (0.966, 0.997)

Metrics are reported with 95% confidence intervals; Abbreviations: AFib, Atrial fibrillation; AUC, Area under the receiver operating characteristic; CD, Conduction disturbance task; LAFB, Left anterior fascicular block; LBBB, Left bundle branch block; LQT, Long QT; Macro-avg, Macro-averaged metric; PACE, Paced rhythm; RBBB, Right bundle branch block; SEN, Sensitivity; SPE, Specificity; STach, Sinus tachycardia; TACHY, Tachycardia task; WPW, Wolff-Parkinson-White pattern.

Supplementary Figure 3. Performance effect of fine-tuning on PTB-XL, on Tachycardia (TACHY – A), Conduction disturbance (CD – B), Long QT (LQT – C), Wolff-Parkinson-White (WPW – D), and Paced rhythm (PACE – E) tasks. Abbreviation: AUROC, Area under the receiver operating characteristic curve.

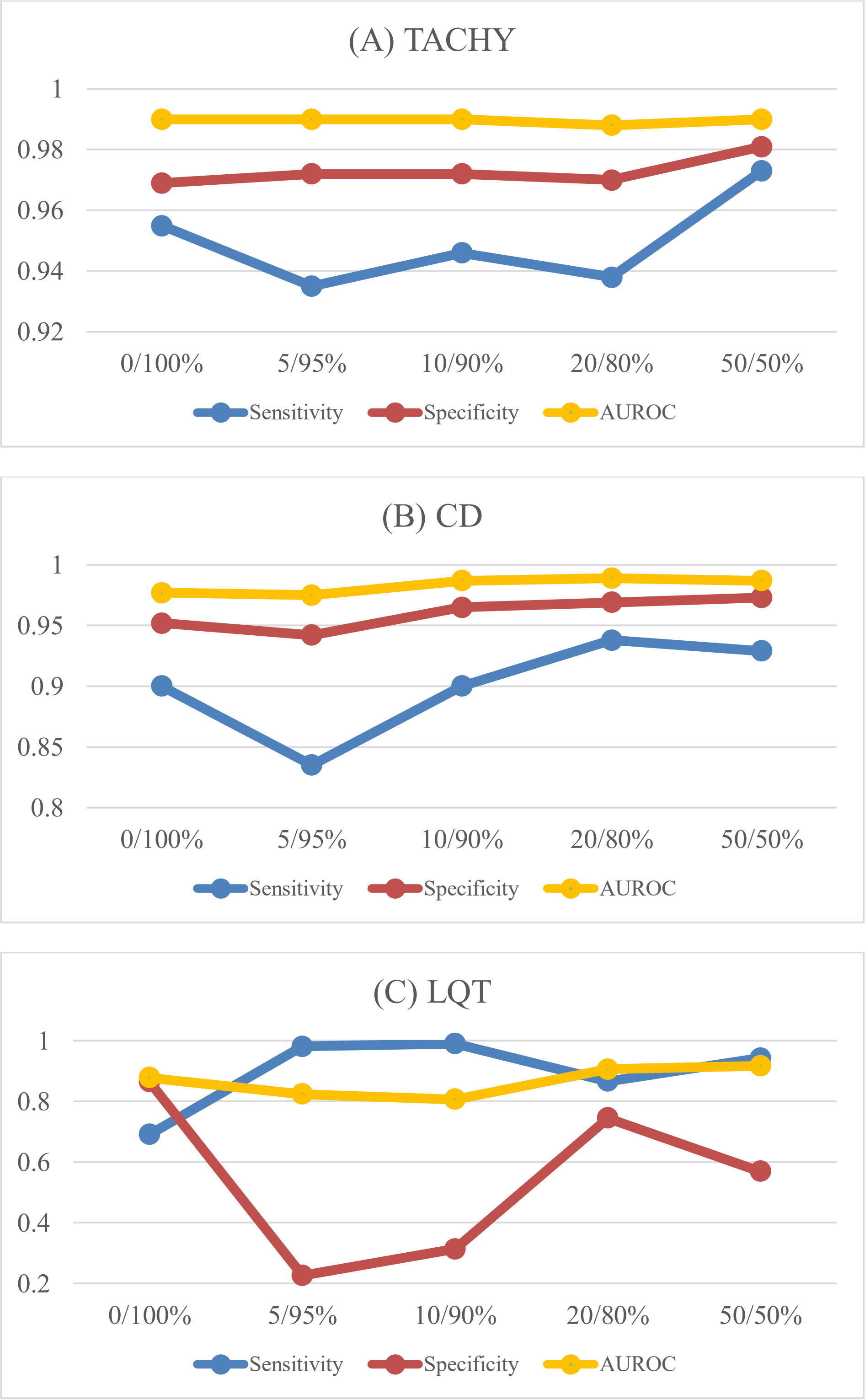

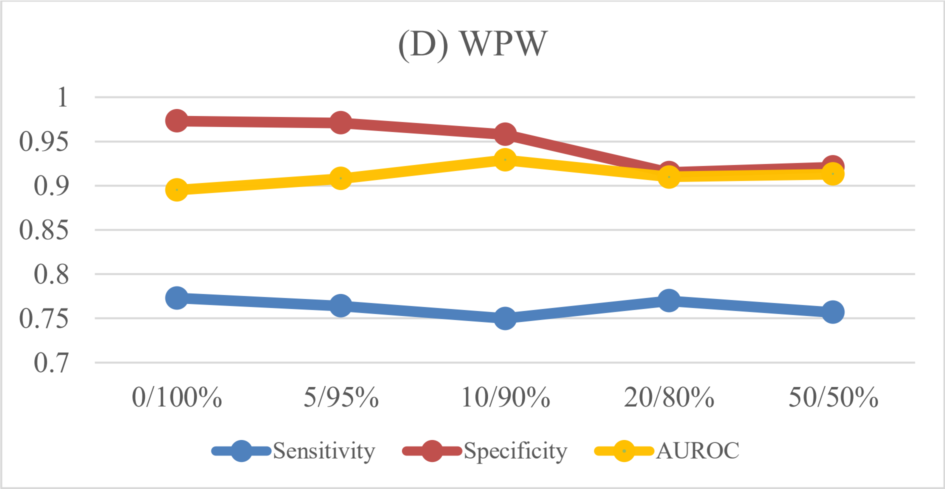

(D) WPW
1
0.95
0.9
0.85
0.8
0.75
0.7
0/100%
5/95%
10/90%
20/80%
50/50%
Sensitivity
Specificity
AUROC

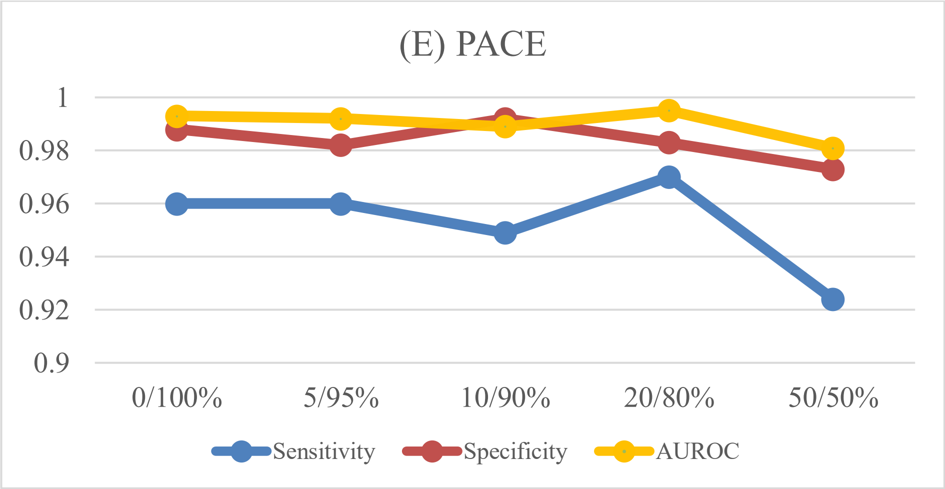

(E) PACE
1
0.98
0.96
0.94
0.92
0.9
0/100%
5/95%
10/90%
20/80%
50/50%
Sensitivity
Specificity
AUROC

7 Threshold Analysis: Sensitivity/Specificity Trade-off

To evaluate the impact of decision thresholds on diagnostic performance, we examined the effect of varying the classification threshold from 0.0 to 1.0 (in increments of 0.1) on macro-averaged sensitivity and specificity for each task. This analysis reflects the trade-off between identifying as many true positives as possible (sensitivity) versus minimizing false positives (specificity).

Key observations:

- TACHY (AFib/STach): Sensitivity remained ≥0.95 until thresholds exceeded 0.5, after which specificity progressively increased at the expense of sensitivity. At a threshold of 0.4–0.5, sensitivity (0.968–0.955) and specificity (0.952–0.969) were both high, suggesting a balanced operating point.
- Conduction disturbances (CD): Sensitivity steadily declined with higher thresholds, while specificity rose, achieving a near-equal trade-off around threshold 0.5 (sensitivity 0.9, specificity 0.952).
- LQT: This task showed the steepest decline in sensitivity with rising thresholds (0.900 at 0.1 vs. 0.473 at 0.8), while specificity increased from 0.609 at 0.1 to 0.936 at 0.8. This highlights the need for sensitivity-oriented thresholds in screening contexts.
- WPW: Performance was more stable, with sensitivity ≥0.75 up to threshold 0.7, accompanied by specificity values >0.95 at thresholds ≥0.5.
- PACE: Sensitivity and specificity both remained high across thresholds, with specificity reaching 0.993 at 0.6 and sensitivity still above 0.94, underscoring robust performance for paced rhythm detection.

These results suggest that ECG-XPLAIM can be adapted for different clinical use cases: lower thresholds for screening (favoring sensitivity) and higher thresholds for diagnostic confirmation (favoring specificity).

Supplementary Table 6. Sensitivity/specificity trade-offs across prediction thresholds (macro-averaged per diagnostic task).

Prediction threshold	*Diagnostic task*									
	TACHY		*CD*		*LQT*		*WPW*		*PACE*	
	SEN	*SPE*	*SEN*	*SPE*	*SEN*	*SPE*	*SEN*	*SPE*	*SEN*	*SPE*
0.0	1.0	0.0	1.0	0.0	1.0	0.0	1.0	0.0	1.0	0.0
0.1	0.998	0.82	0.949	0.907	0.9	0.609	0.8	0.88	0.986	0.848
0.2	0.983	0.898	0.937	0.924	0.864	0.736	0.787	0.907	0.969	0.941

0.3	0.976	0.932	0.925	0.934	0.818	0.773	0.783	0.913	0.969	0.972
0.4	0.968	0.952	0.912	0.941	0.718	0.8	0.779	0.953	0.959	0.983
0.5	0.955	0.969	0.9	0.952	0.691	0.864	0.773	0.973	0.96	0.988
0.6	0.938	0.976	0.886	0.956	0.582	0.877	0.768	0.978	0.942	0.993
0.7	0.909	0.982	0.86	0.961	0.509	0.888	0.754	0.978	0.931	0.997
0.8	0.86	0.988	0.829	0.965	0.473	0.936	0.701	0.982	0.917	0.997
0.9	0.768	0.992	0.767	0.974	0.355	0.982	0.62	0.987	0.881	0.999
1.0	0.0	1.0	0.03	1.0	0.0	1.0	0.0	1.0	0.0	1.0

Values ≥0.90 are highlighted in orange, and values ≥0.95 in green. Abbreviations: AUC, Area under the receiver operating characteristic curve; CD, Conduction disturbance task; LQT, Long QT task; PACE, Paced rhythm task; SEN, Sensitivity; SPE, Specificity; TACHY, Tachycardia task; WPW, Wolff-Parkinson-White pattern task.

8 Explainability Analysis with Grad-CAM

We present a selection of correctly (see 4.1) and falsely (see 4.2) classified cases to illustrate how the model's decision-making aligns with clinical criteria and where potential biases or misinterpretations arise. For correctly classified cases, ECG-XPLAIM successfully highlighted expected waveform regions associated with each arrhythmia, demonstrating alignment with known diagnostic features. For falsely classified cases, we observed instances where the model's attention was misdirected, revealing possible biases in training and suggesting areas for improvement. By examining these visualizations, we gain deeper insights into the model's strengths and limitations, helping to guide refinements in dataset distribution and training strategies.

8.1 Correctly classified samples (selected cases)

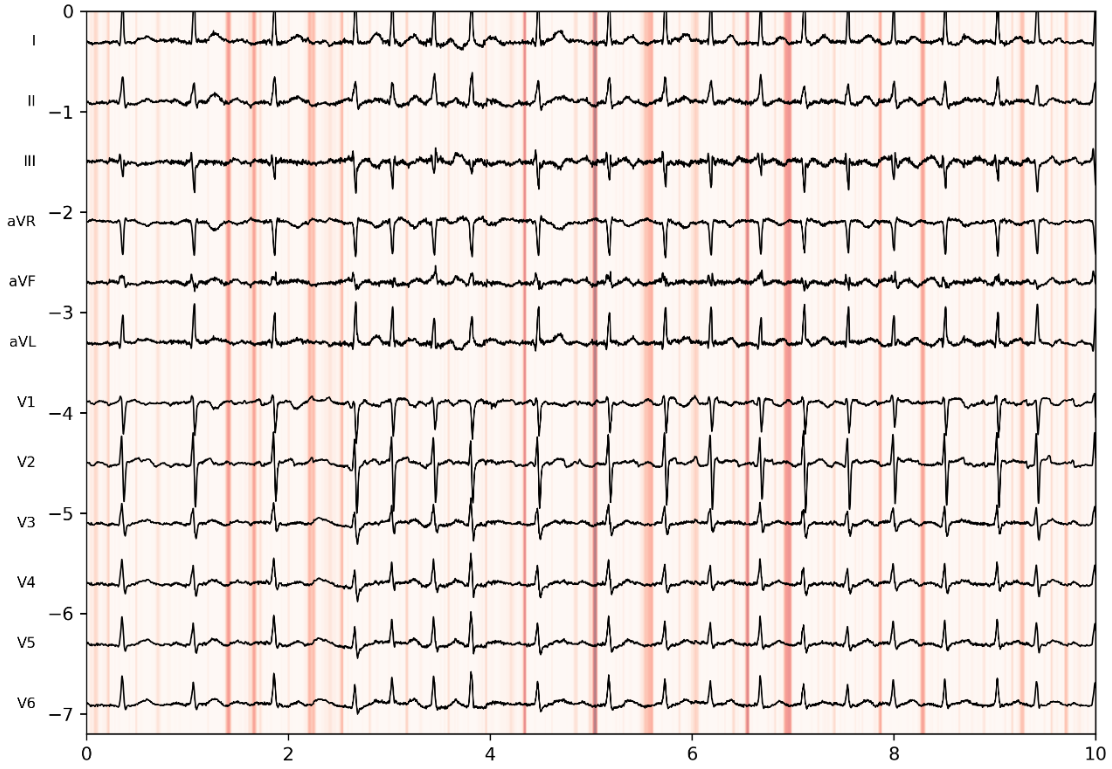

Supplementary Figure 4. Correctly classified atrial fibrillation (AFib) case (true positive). ECG-XPLAIM highlights pre-QRS regions, correctly identifying the absence of P waves as a key distinguishing feature.

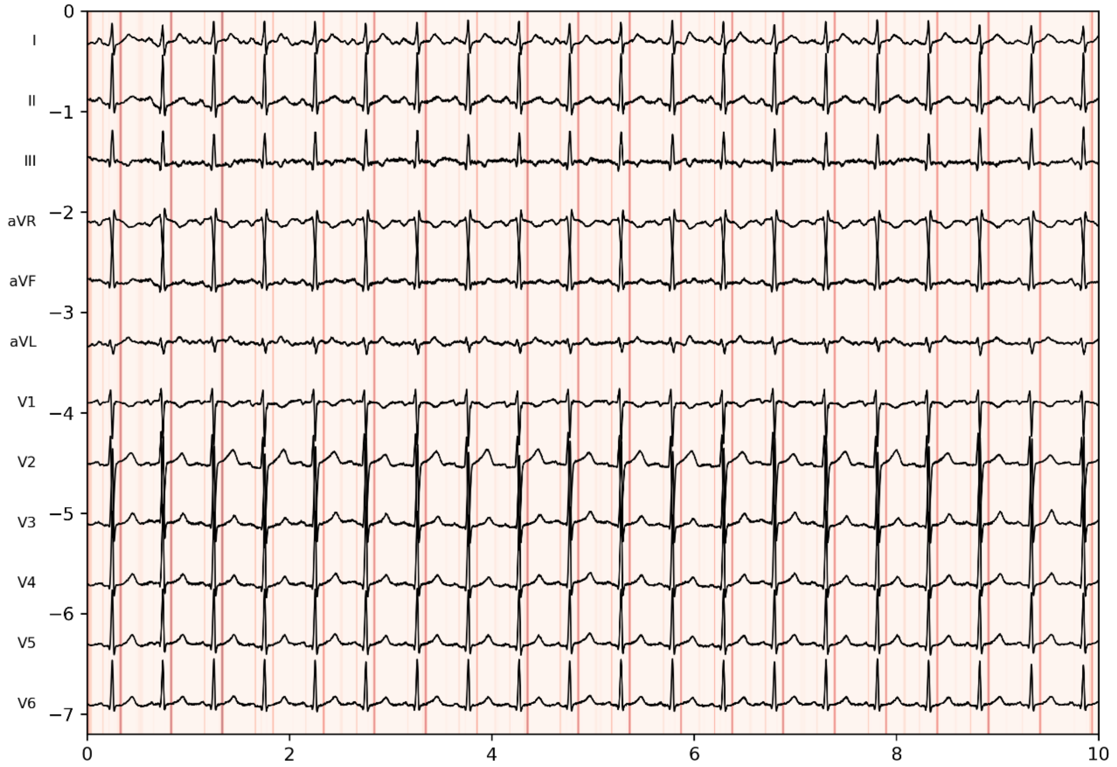

Supplementary Figure 5. Correctly classified sinus tachycardia (STach) case (true positive). ECG-XPLAIM highlights pre-QRS regions, focusing on P-wave morphology, along with a segment of the ST interval, potentially indicating the model's emphasis on rhythm regularity.

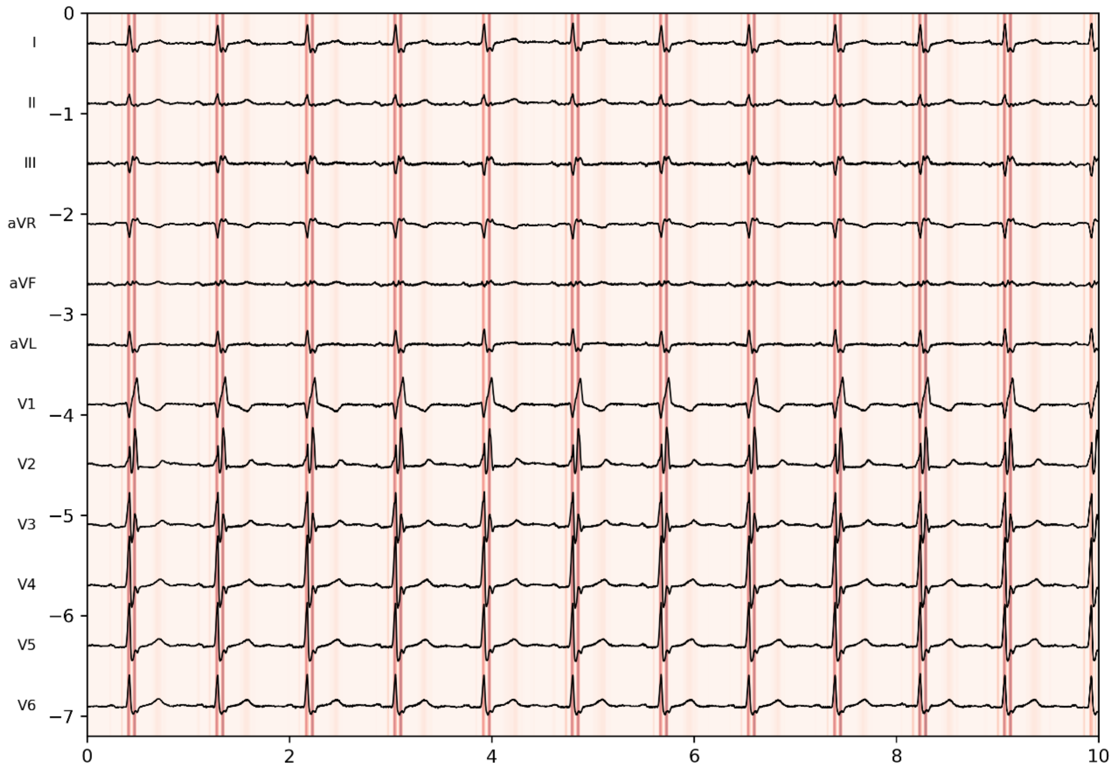

Supplementary Figure 6. Correctly classified right bundle branch block (RBBB) case (true positive). ECG-XPLAIM highlights the QRS complex, with a pronounced focus on the R peak in leads V1–V2, aligning with characteristic RBBB morphology.

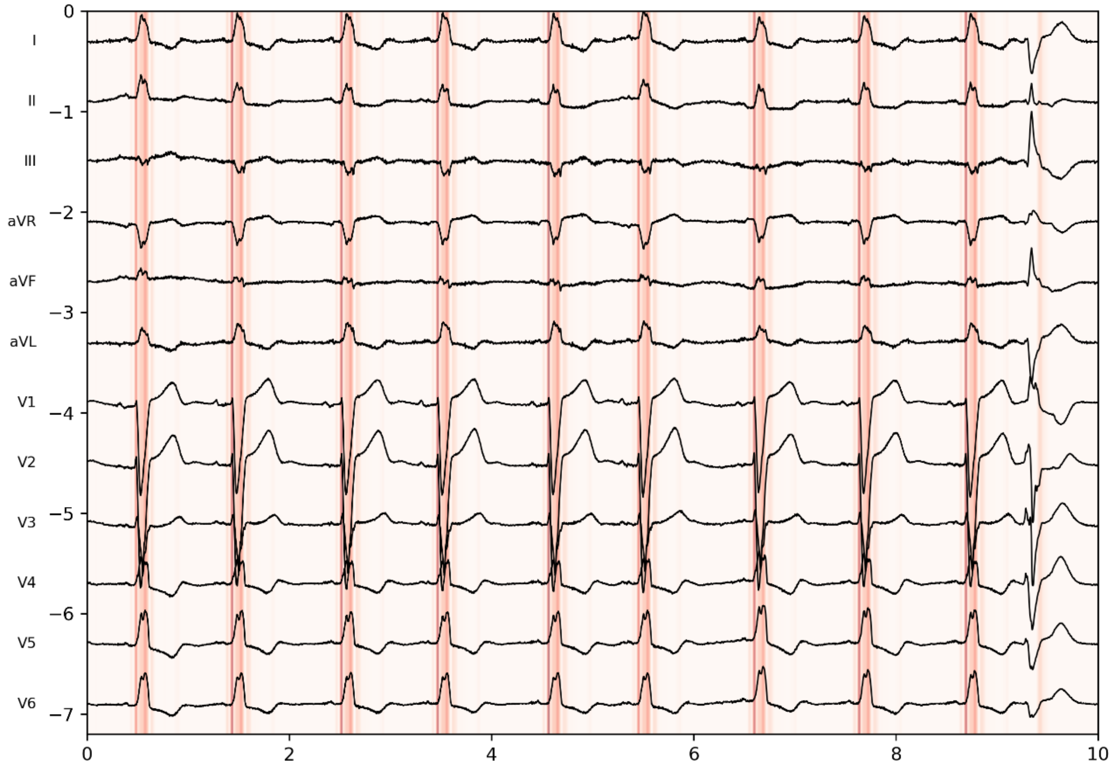

Supplementary Figure 7. Correctly classified left bundle branch block (LBBB) case (true positive). ECG-XPLAIM emphasizes the entire QRS complex, its prolonged duration, and morphology, aligning with the characteristic features of LBBB.

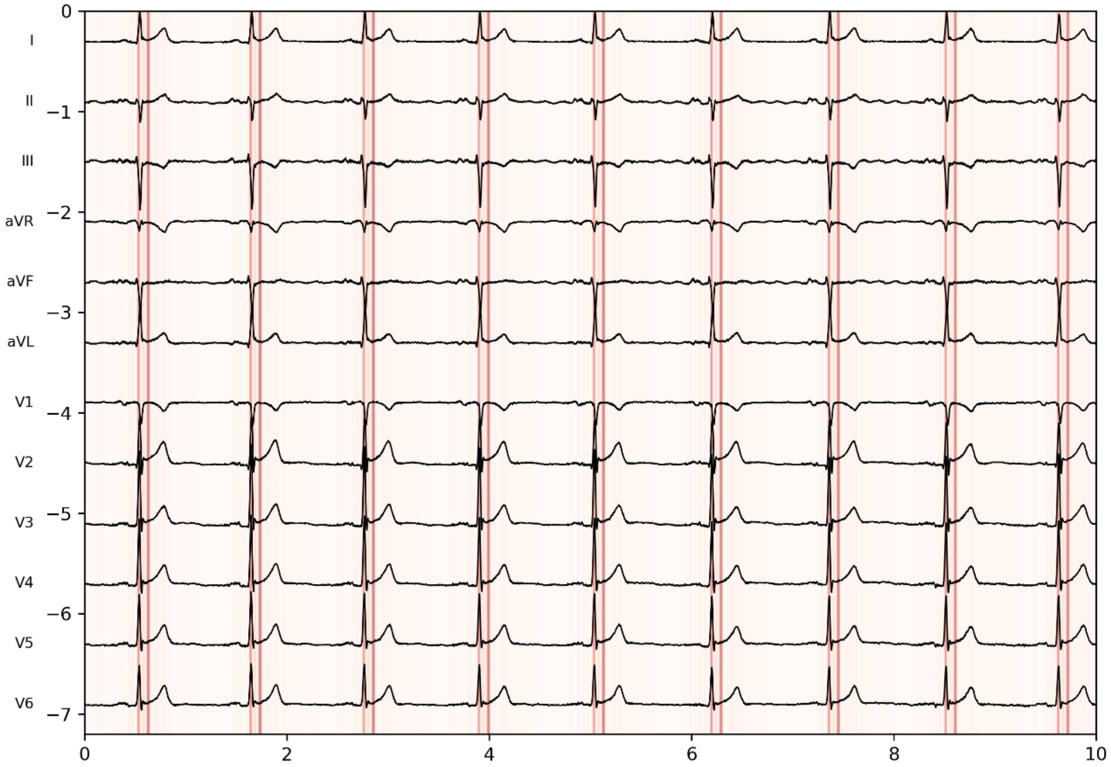

Supplementary Figure 8. Correctly classified left anterior fascicular block (LAFB) case (true positive). ECG-XPLAIM highlights the QRS complex, likely capturing axis deviation associated with LAFB.

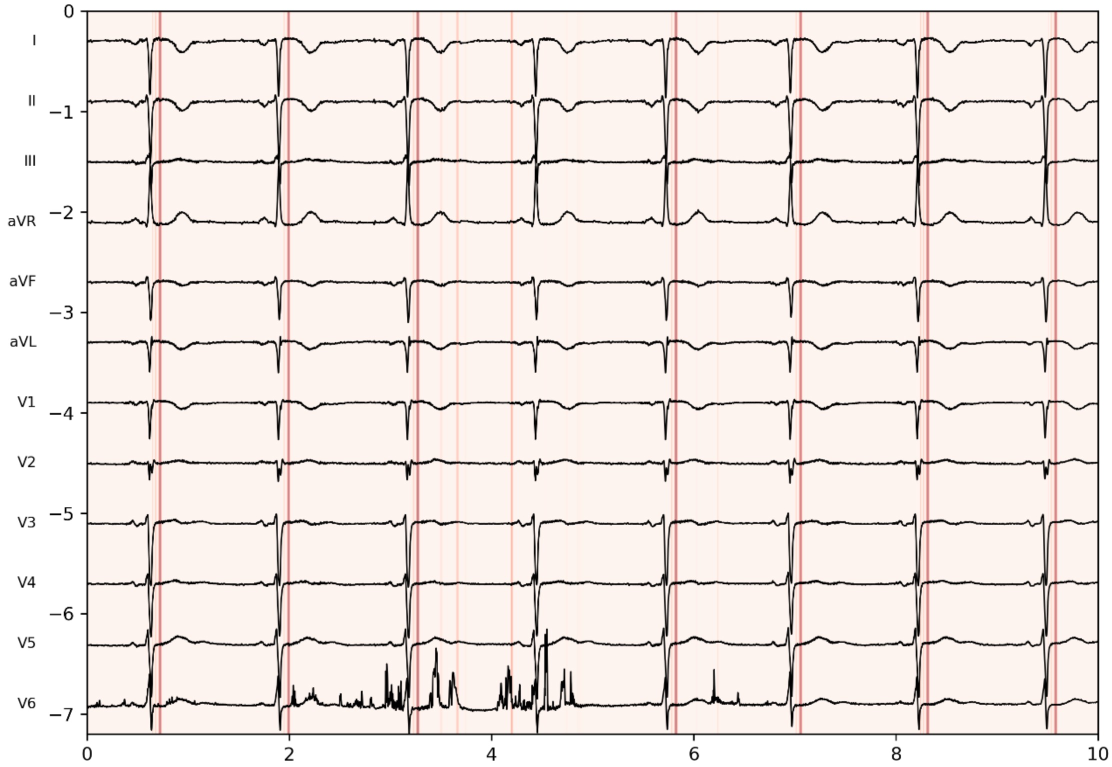

Supplementary Figure 9. Correctly classified long QT (LQT) case (true positive). ECG-XPLAIM highlights regions closely to the onset and termination of the QT interval in some beats, reflecting its focus on QT duration.

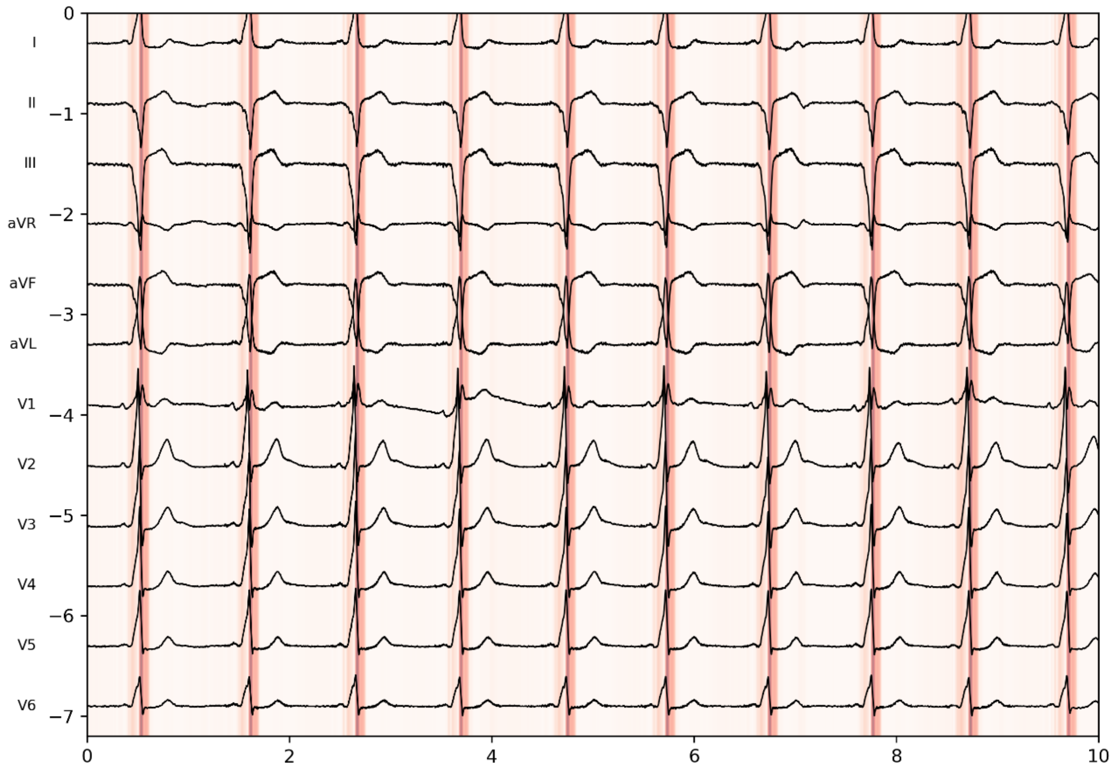

Supplementary Figure 10. Correctly classified Wolff-Parkinson-White (WPW) case (true positive). ECG-XPLAIM highlights the terminal portion of the QRS complex, as well as the segment between the end of the P-wave and the R-peak, aligning with the characteristic delta wave and interval alterations seen in WPW.

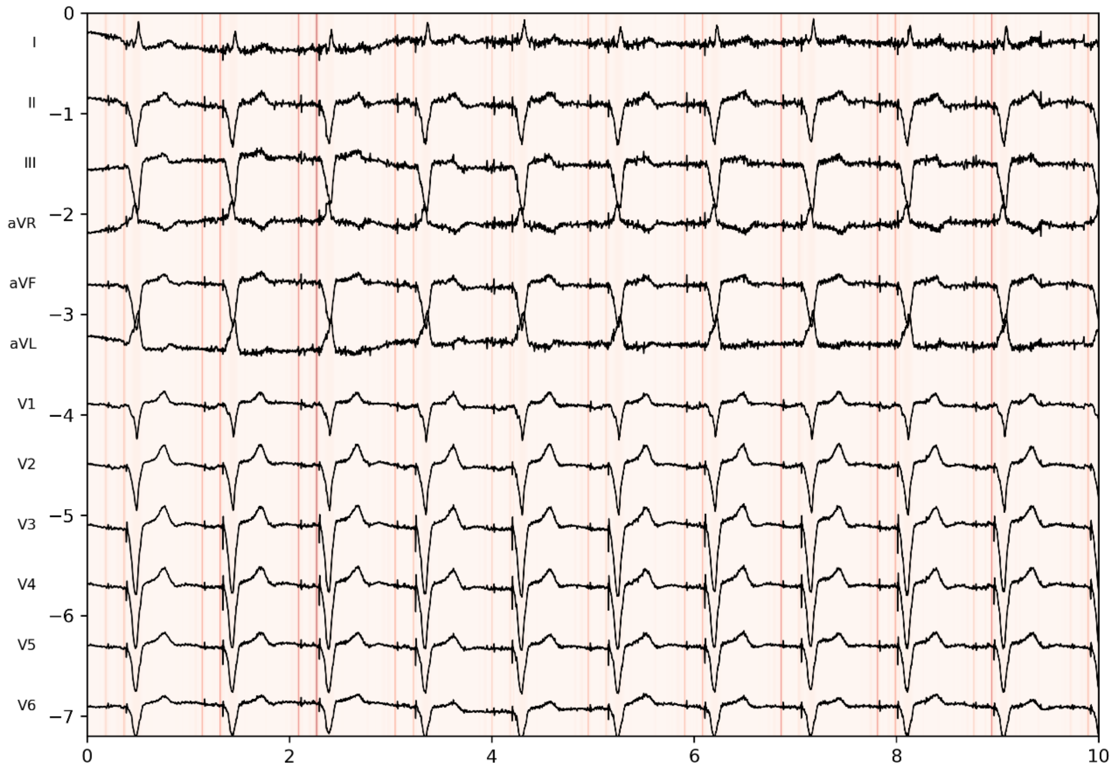

Supplementary Figure 11. Correctly classified paced rhythm (PACE) case (true positive). ECG-XPLAIM focuses on both atrial and ventricular pacing spikes across all beats, indicating pacemaker activity.

8.2 Falsely classified samples (selected cases)

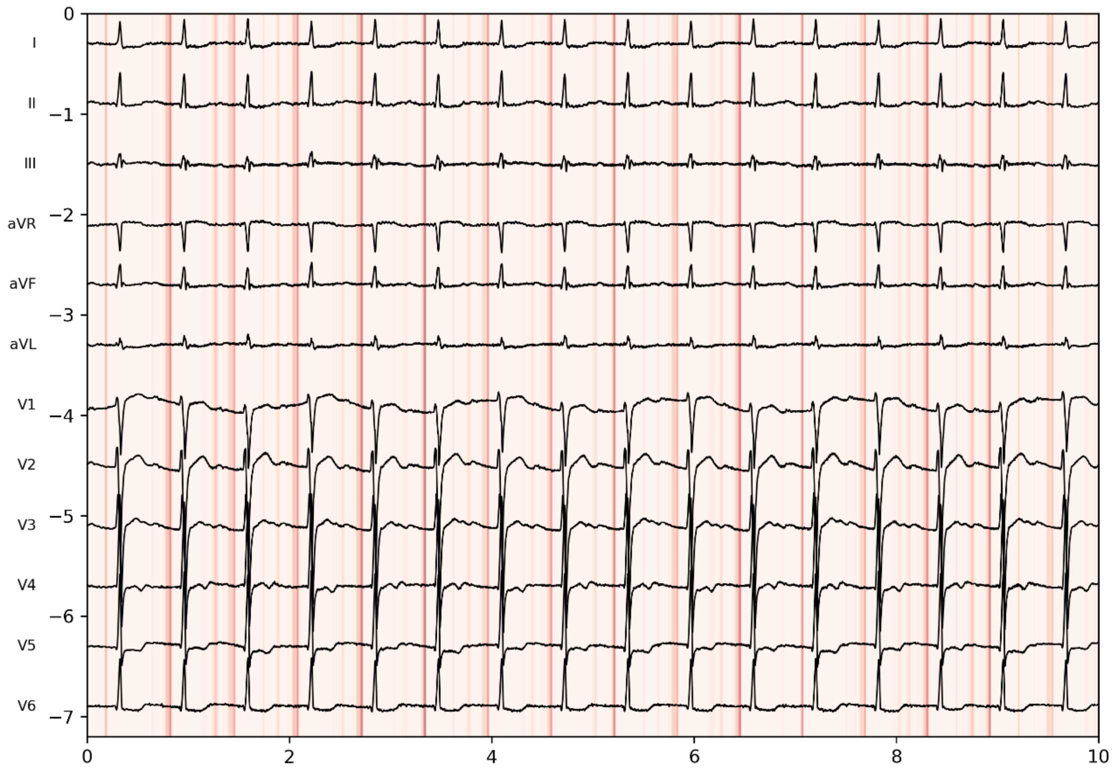

Supplementary Figure 12. False positive atrial fibrillation (AFib) classification. ECG-XPLAIM focuses on the pre-QRS region, where the P-wave is typically expected. However, in this case, the P-wave appears earlier due to first-degree AV block (I-AVB), leading to misclassification. This suggests a model bias in P-wave localization, highlighting the need for additional training on conduction abnormalities such as I-AVB.

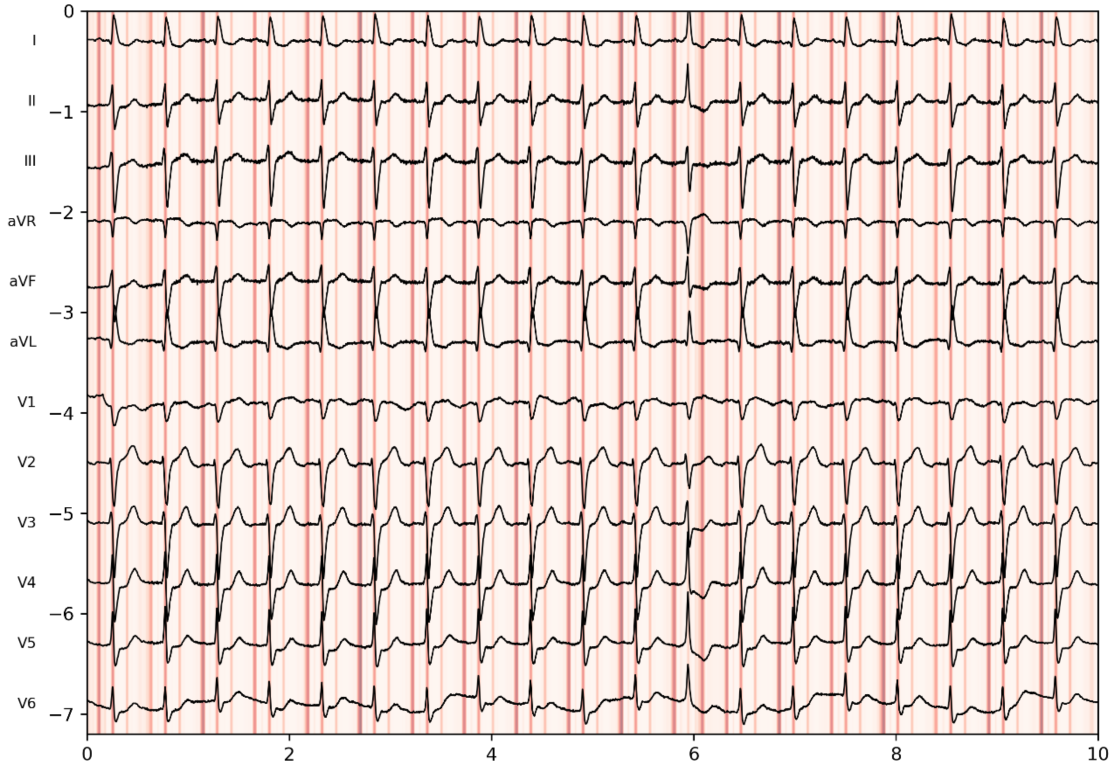

Supplementary Figure 13. False negative sinus tachycardia (STach) classification, but also false positive for atrial fibrillation (AFib). ECG-XPLAIM correctly detects tachycardia, possibly based on the QRS rhythmicity, but misclassifies STach as AFib, likely due to its focus on the pre-QRS region where P-waves are expected. In this case, the P-waves are of lower amplitude and nearly fused with the preceding T-wave due to tachycardia, potentially confusing the model. This suggests a need for further training on high-rate rhythms with subtle P-wave visibility.

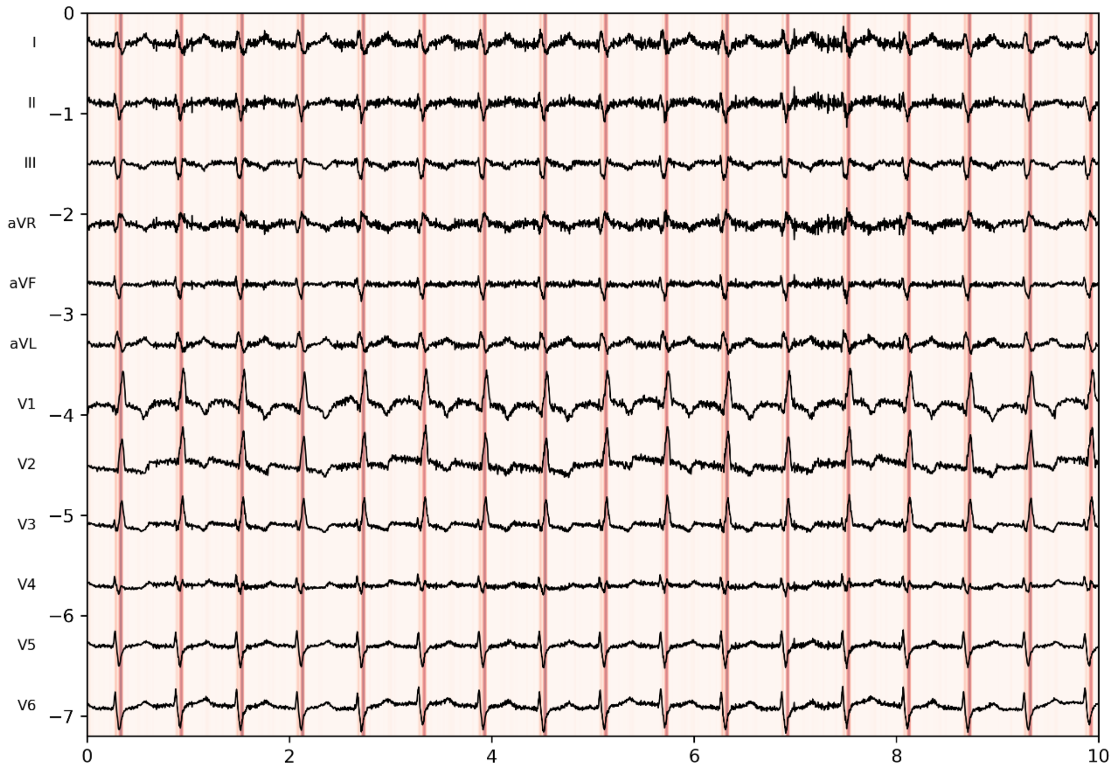

Supplementary Figure 14. False negative right bundle branch block (RBBB) classification. ECG-XPLAIM primarily focuses on the QRS complex, particularly the R-peak, but fails to classify the ECG as RBBB, possibly due to a relatively shorter QRS duration compared to more pronounced RBBB cases, suggesting that the model may require additional training on borderline cases.

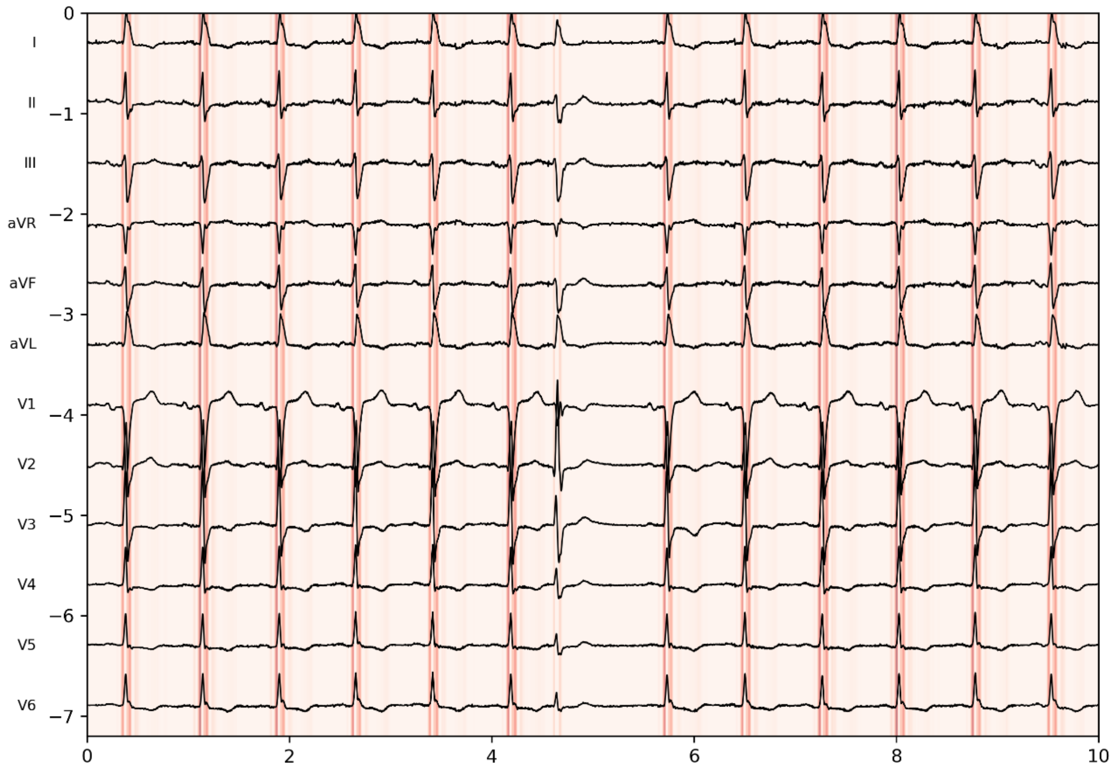

Supplementary Figure 15. False negative left bundle branch block (LBBB) classification. ECG-XPLAIM focuses on the QRS complex but fails to classify the ECG as LBBB, likely due to a relatively shorter yet still pathological QRS duration.

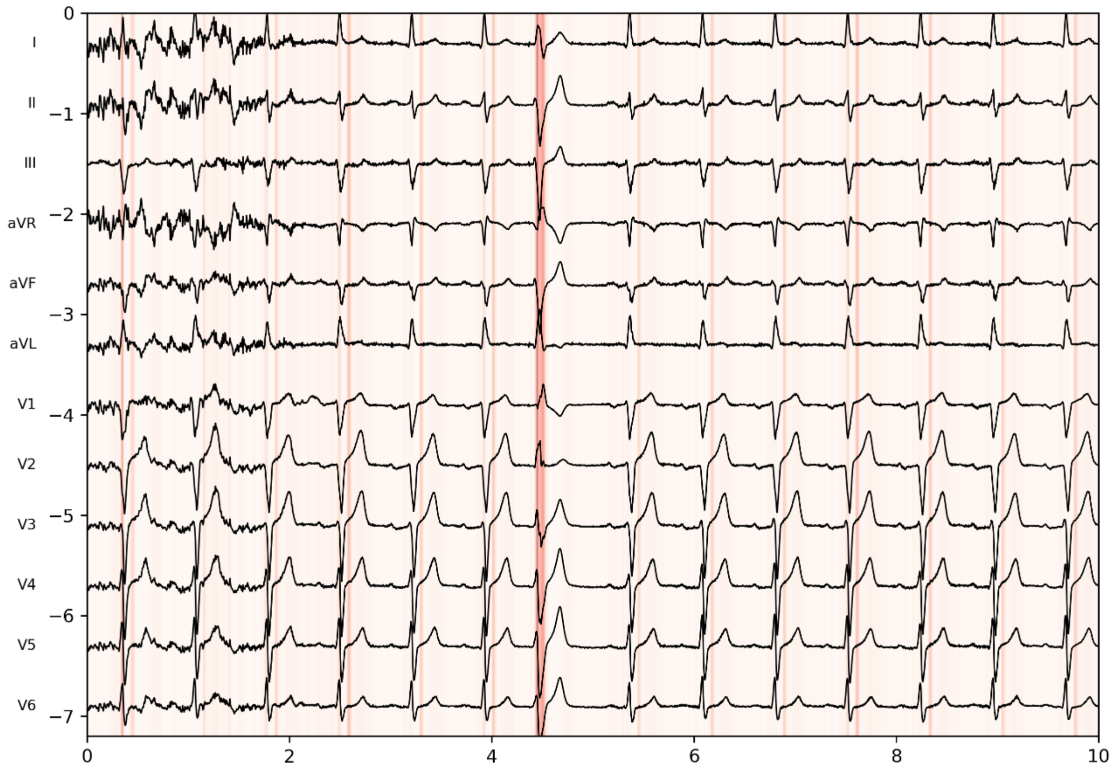

Supplementary Figure 16. False positive left anterior fascicular block (LAFB) classification. ECG-XPLAIM misinterprets a premature beat, likely drawing conclusions based on axis deviations associated with the extrasystole rather than the overall rhythm.

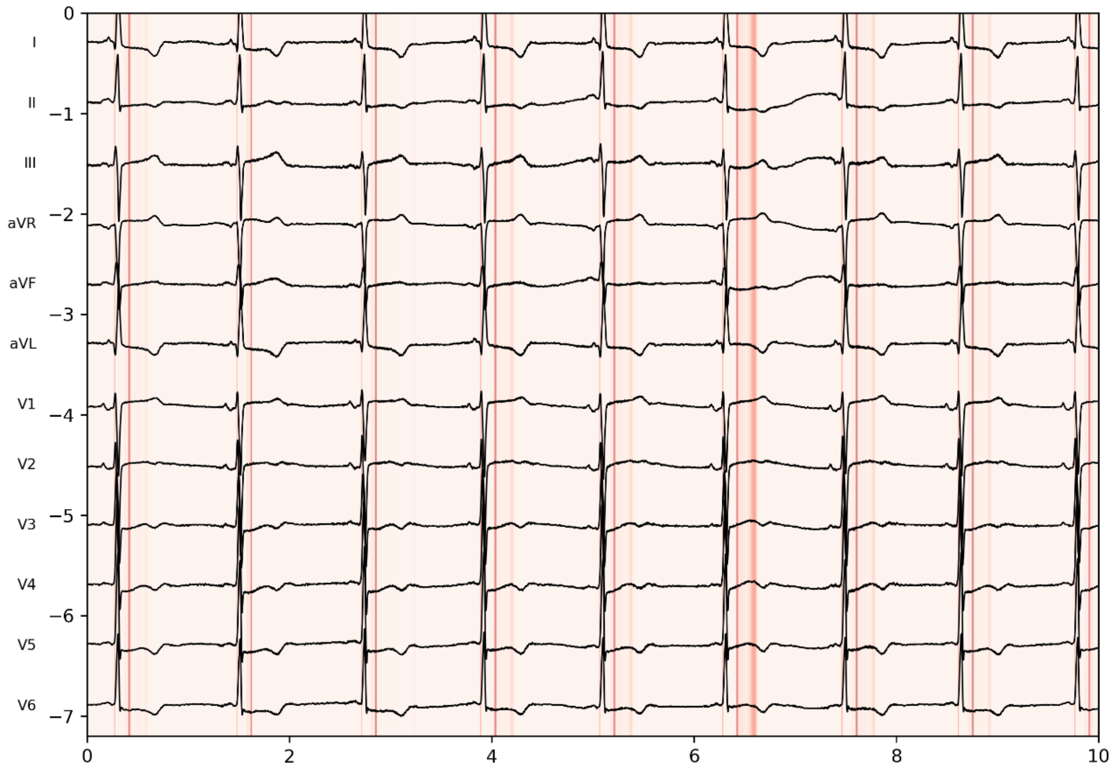

Supplementary Figure 17. False negative long QT (LQT) classification. ECG-XPLAIM highlights a segment within the QT interval rather than its full duration, potentially leading to misclassification.

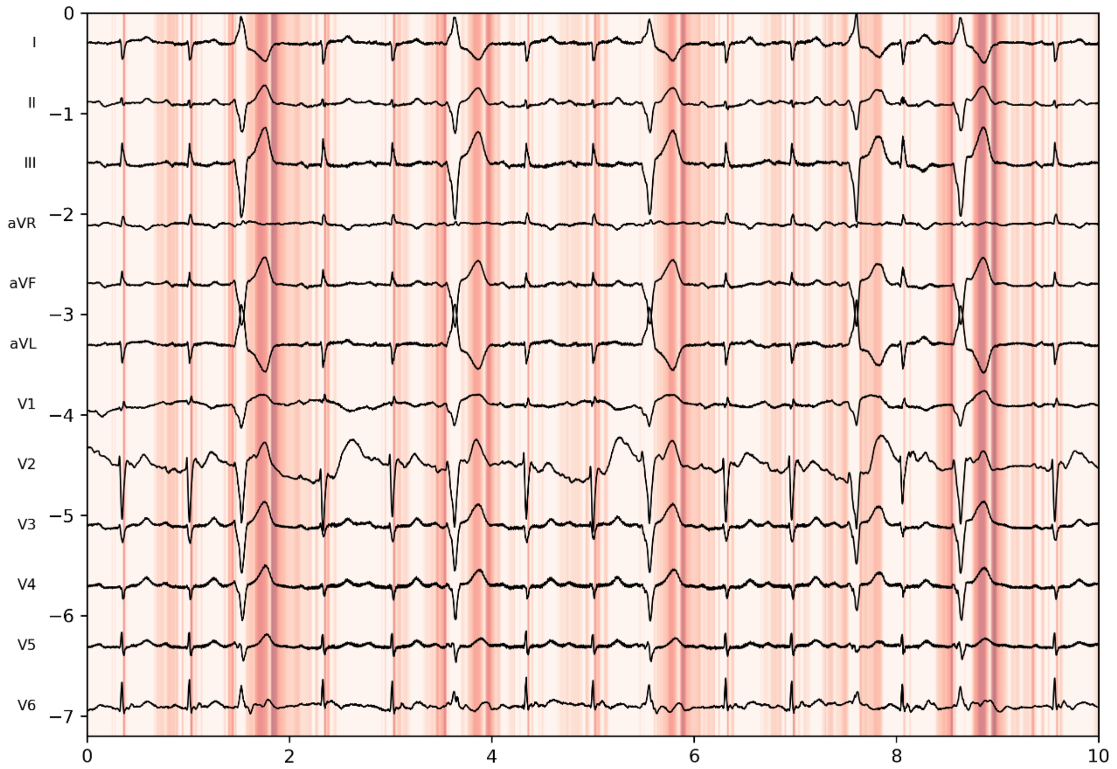

Supplementary Figure 18. False positive Wolff-Parkinson-White (WPW) classification. ECG-XPLAIM places significant focus on the pre-QRS regions of some wide-QRS extrasystoles, potentially mistaking premature beat morphology for delta waves. This suggests a bias toward pre-QRS intervals in abnormal beats, highlighting the need for additional training on differentiating true delta waves from misleading distortions.

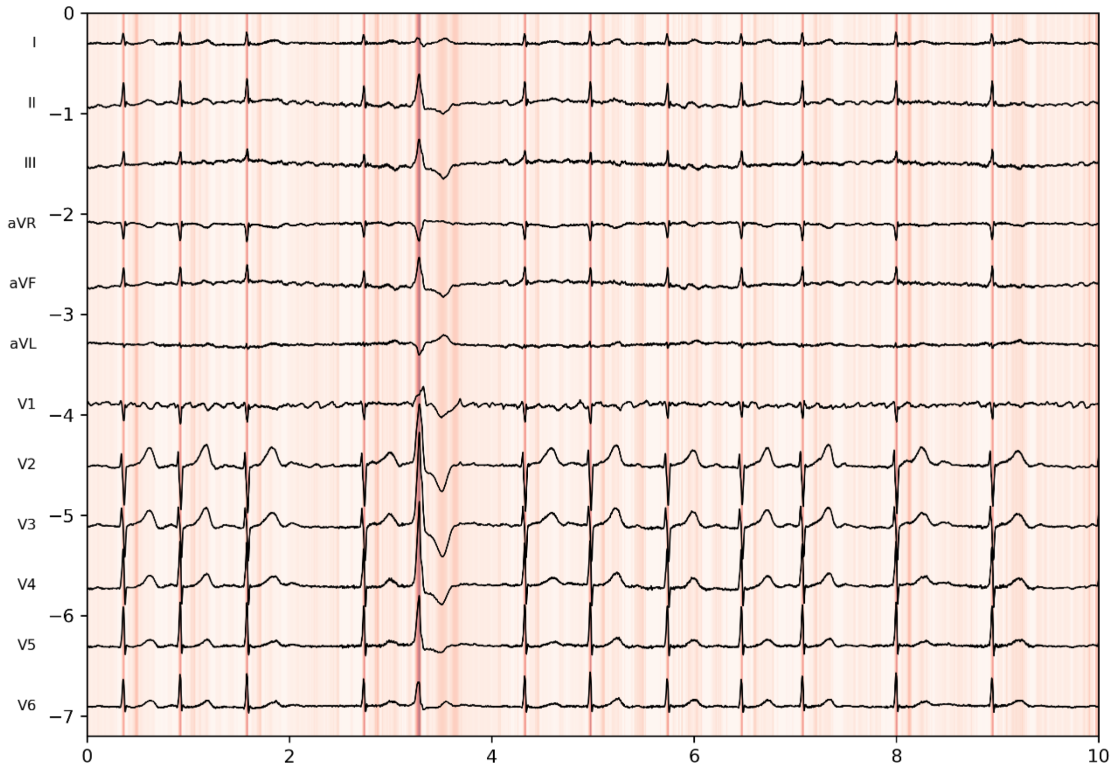

Supplementary Figure 19. False positive pacemaker (PACE) classification. ECG-XPLAIM does not detect pacing spikes in most beats but misclassifies the ECG as paced, likely due to its focus on a premature complex and the preceding narrow, regular QRS.
